# Proteasome inhibition alleviates proteinuria in *Lmx1b* knock-in mice with dysfunctional LIM domains

**DOI:** 10.1038/s41467-026-77485-1

**Published:** 2026-09-11

**Authors:** Joshua Hermens, Lisa Lucke, Oliver Pieles, Olga Maier, Helga Othmen, Tillmann Burghardt, Alexander Schmidt, Markus Moser, M. Gregor Madej, Uwe Schwartz, Melanie Zaparty, Ralph Witzgall

**Affiliations:** 1 Institute for Molecular and Cellular Anatomy, University of Regensburg, Regensburg, Germany; 2 Max-Planck-Institute of Biochemistry, Department of Molecular Medicine, Martinsried, Germany; 3 Institute of Experimental Hematology, Technische Universität München, Munich, Germany; 4 Department of Biophysics, University of Regensburg, Regensburg, Germany; 5 NGS Analysis Center, Faculty of Biology and Pre-Clinical Medicine, University of Regensburg, Regensburg, Germany

**Keywords:** Glomerular diseases, Experimental models of disease, Medical genetics

## Abstract

Mutations in the transcription factor LMX1B have been identified as the cause of the autosomal-dominant disease nail-patella syndrome. It manifests in small or absent patellae and dysplastic or missing toe- and fingernails, but the prognosis of the patients is determined by the development of renal symptoms due to dysfunctional podocytes. The pathogenetic mechanisms leading to podocyte damage and their association with specific mutations in the *LMX1B* gene are not understood, which has impeded the development of specific therapeutic strategies. Here we identify the pathogenetic mechanism affecting many patients with nail-patella syndrome, provide proof of principle for a novel therapeutic approach and suggest a domain-specific effect in gene regulation by LMX1B. Interestingly, missense mutations in the LIM domains of Lmx1b result in a recessive phenotype in two different knock-in mouse lines due to proteasomal degradation of the mutated proteins. The decreased half-life of the mutated LMX1B proteins can be attributed to ubiquitinylation and is prolonged by two proteasomal inhibitors, one of which was also tested in our mouse models and results in an alleviation of the renal symptoms. RNA sequencing of genetically altered podocytes indicates that LMX1B predominantly acts as a transcriptional repressor and furthermore suggests that the two LIM domains contribute to the distinct regulation of LMX1B target genes. We conclude that proteasomal inhibitors, drugs already approved for treatment of patients with multiple myeloma, warrant further studies to prevent renal failure in patients with nail-patella syndrome caused by mutations in the LIM domains. These studies should be designed to deliver the drug specifically to podocytes and to target specific ubiquitin ligases in order to limit side effects.

## Abstract (web summary)

Mutations in the LIM domains of LMX1B cause ubiquitin-mediated proteasomal degradation and result in podocyte injury. Inhibition of the proteasome ameliorated renal symptoms in Lmx1b knock-in mice, thus providing proof of principle for a novel therapeutic strategy.

## Introduction

Patients suffering from nail-patella syndrome typically present with malformed fingernails and absent or hypoplastic kneecaps. The prognosis of the patients, however, is determined by a characteristic nephropathy which is caused by a malfunction of the podocytes^1^. Nail-patella syndrome is caused by mutations in the gene *LMX1B,* which encodes a transcription factor of the LIM-homeodomain family^2,3^. A multitude of mutations in the *LMX1B* gene have been described affecting the two LIM domains at the NH_2_-terminus of LMX1B, which are believed to mediate interactions with other proteins, and the DNA-binding homeodomain in the middle of the protein^4^. Although nail-patella syndrome is inherited in an autosomal-dominant fashion, data from genetically manipulated mice in which parts of the *Lmx1b* gene were deleted do not reflect this mode of inheritance. Conventional *Lmx1b* knock-out mice only develop symptoms resembling nail-patella syndrome in a homozygous state^2,5,6^. Furthermore, both the constitutive^7^ and the inducible^8^ podocyte-specific inactivation of *Lmx1b* requires the inactivation of both *Lmx1b* alleles before proteinuria, a hallmark of podocyte damage, can be observed. Possible explanations for the discrepancy between the dominant inheritance in humans and the recessive phenotype in mice have remained a matter of speculation so far. For one, renal symptoms occur in approximately 40% of patients and may take decades to unfold^9^ which could be due to the accumulation of damaged podocytes over the years. Such podocyte injury may result from environmental or genetic causes, such as the somatic mutations (two-hit mechanism) causing cyst formation in autosomal-dominant polycystic kidney disease^10^. Probably the life expectancy of a mouse is too short for such a mechanism to be observed. Furthermore, the somatic injury model cannot explain the fact that the patella defects and nail deformities are already present during gestation, i.e., as soon as the limbs are formed. It must be kept in mind that the mutant alleles used in mice probably represent null alleles, whereas in patients many missense mutations have been found, which may confer a gain of function or a dominant-negative effect.

We decided to test this hypothesis by generating *Lmx1b* knock-in mice which carry the same single amino acid mutations in the first and second LIM domains, respectively, as those described in patients. Such mutations are predicted to disrupt the interaction between LMX1B and necessary co-factors such as LDB1. LDB1 has long been recognized to not only interact with LMX1B, but podocyte-specific *Ldb1* knock-out mice also develop severe proteinuria^7^. Remarkably, heterozygous *Lmx1b*^+/H77L^ and *Lmx1b*^+/C118F^ knock-in mice were healthy and showed no obvious renal and skeletal phenotypes. The phenotype of homozygous *Lmx1b*^H77L/H77L^ and *Lmx1b*^C118F/C118F^ knock-in mice resembled the phenotype of conventional *Lmx1b*^-/-^ knock-out mice. We discovered that the LMX1B^H77L^ and LMX1B^C118F^ mutant proteins are unstable and show a reduced half-life compared to wild-type LMX1B. Consequently, we generated two mouse lines in which one Lmx1b allele contained the respective missense mutation and the other *Lmx1b* allele was floxed. The doxycycline-inducible induction of Cre recombinase specifically in podocytes resulted in the inactivation of the floxed allele and in pronounced proteinuria after seven days of induction. Through the administration of the proteasomal inhibitor bortezomib we were able to block the degradation of the mutant proteins both in vitro and in vivo and thereby alleviated the renal phenotype of both the compound *Lmx1b*^H77L/lox^ and the compound *Lmx1b*^C118F/lox^ knock-in lines. Therefore, proteasomal inhibition may be the first therapeutic approach for treating the renal symptoms of patients with nail-patella syndrome.

## Results

### Only homozygous Lmx1b^H77L^ and Lmx1b^C118F^ knock-in mice present with an obvious phenotype

**Fig. 1:**
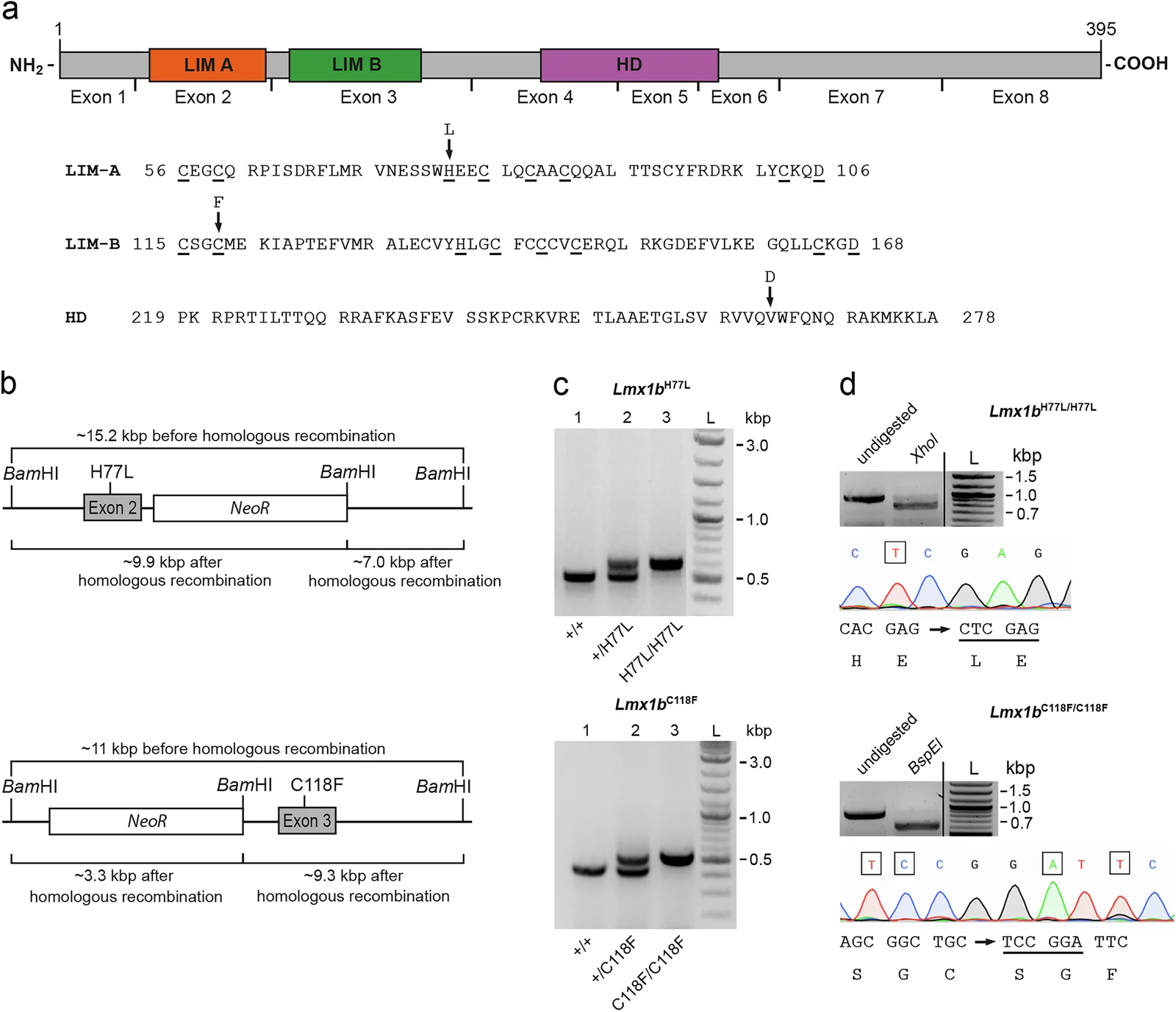
Generation of *Lmx1b*^H77L^ and *Lmx1b*^C118F^ knock-in mice. **a** The cartoon shows the exon structure of the human *LMX1B* gene and the corresponding domain organization of the protein. Below the cartoon the amino acids comprising the LIM-A and -B domains and the homeodomain are shown, zinc-coordinating residues are underlined (human and murine sequences are identical, numbering according to the human protein as given in the sequence with accession number NP_002307.2). The position of the missense mutations is indicated by arrows. **b** Shown are the respective portions of the murine *Lmx1b* gene after homologous recombination of the targeting construct employed for the generation of the knock-in mice. *NeoR*, neomycin resistance cassette flanked by FRT sites. **c** PCR of genomic DNA isolated from tail biopsies was performed with the appropriate primers. The PCR products from wild-type mice are shorter (493 bp and 428 bp, respectively) than the PCR products from the respective knock-in mice (585 bp and 526 bp, respectively) due to the presence of a remaining FRT site after FLP-mediated deletion of the neomycin resistance cassette. This pattern was routinely confirmed upon the genotyping of offspring. **d** PCR of reverse-transcribed RNA isolated from total embryos of the respective homozygous knock-in mice at embryonic day 18.5 yielded a PCR product of 959 bp. *Xho*I and *Bsp*EI sites (underlined), respectively, were introduced together with the amino acid-altering point mutations (highlighted by squares) in the respective codons as can be seen from the higher mobility of the digested fragments. Sequencing of the PCR products confirmed the presence of the desired mutations. Three mice were analyzed for each genotype. Source data are provided as a Source Data file.

The classical Cys_4_ and Cys_2_His_2_ zinc finger motifs contain four zinc-coordinating amino acids and mediate binding to DNA, whereas LIM domains with the consensus sequence CysX_2_CysX_16-23_HisX_2_CysX_2_CysX_2_CysX_16-21_CysX_2_Cys/His/Asp^11,12^ are characterized by the presence of eight zinc-coordinating amino acids, and they are responsible for the interaction with other proteins. Amino acid substitutions affecting the zinc-coordinating residues of LMX1B have been made responsible for the development of nail-patella syndrome^4^. In order to investigate the effect of missense mutations, we generated targeting constructs in which either exon 2 encoding the LIM-A or exon 3 encoding the LIM-B domain carried an amino acid-altering mutation (Fig. 1a). The corresponding mutant proteins, LMX1B^H77L^ and LMX1B^C118F^ (numbering according to the 395-amino acid long LMX1B protein^13^), have been described in patients with nail-patella syndrome but not in healthy individuals and are therefore known disease-causing variants of LMX1B (described as H54L and C95F in reference Bongers et al.^4^). Integration of the mutated embryonic stem cells into the germ line was confirmed by PCR and sequencing (Fig. 1b–d). Contrary to our expectations, however, we did not detect any overt phenotypic alterations in heterozygous *Lmx1b*^+/H77L^ and *Lmx1b*^+/C118F^ mice even after careful morphological examination (see below, Supplementary Figs. 1, 2), and we also did not observe a reduced life span. This suggested that the H77L and C118F mutant proteins did not act in a dominant-negative fashion or as gain-of-function mutants but rather represented loss-of-function mutants. To further corroborate that the H77L and C118F mutations resulted in a loss of function, we crossed both *Lmx1b* knock-in lines to homozygosity to obtain *Lmx1b*^H77L/H77L^ and *Lmx1b*^C118F/C118F^ mice. Since this led to perinatal lethality, it supported the notion that the H77L and C118F mutations disrupted the structure of the LIM-A and LIM-B domains, respectively, and led to a loss of function in either case.

Conventional homozygous *Lmx1b* knock-out mice (*Lmx1b*^-/-^ mice) suffer from skeletal defects, specifically they lack patellae and develop foot pads not only on the ventral but also on the dorsal side of their paws^2^ which attests to the role of LMX1B in dorso-ventral axis formation during limb development^14,15^. In light of the fact that missense mutations in the LIM-A and LIM-B domains have been detected in patients with nail-patella syndrome, we investigated whether the homozygous *Lmx1b*^H77L/H77L^ and *Lmx1b*^C118F/C118F^ mice also suffer from skeletal defects. The presence and absence of patellae could not be assessed unequivocally but we also noticed that the distal ulna was missing (Supplementary Fig. 1) as reported before for the *Lmx1b*^-/-^ mice^2^. In addition, μCT images demonstrated abnormal calvarial structures (Supplementary Fig. 2) again as described in the conventional *Lmx1b* knock-out mice^16^. Finally, E18.5 embryos from both homozygous *Lmx1b* knock-in lines also formed foot pads on the dorsal side (Supplementary Fig. 3). Therefore we conclude that, for either LIM domain, homozygous knock-in mice present with skeletal defects indistinguishable from those of the homozygous *Lmx1b* knock-out mice whereas heterozygous knock-in mice, like the heterozygous knock-out mice, do not develop an obvious phenotype.

**Fig. 2:**
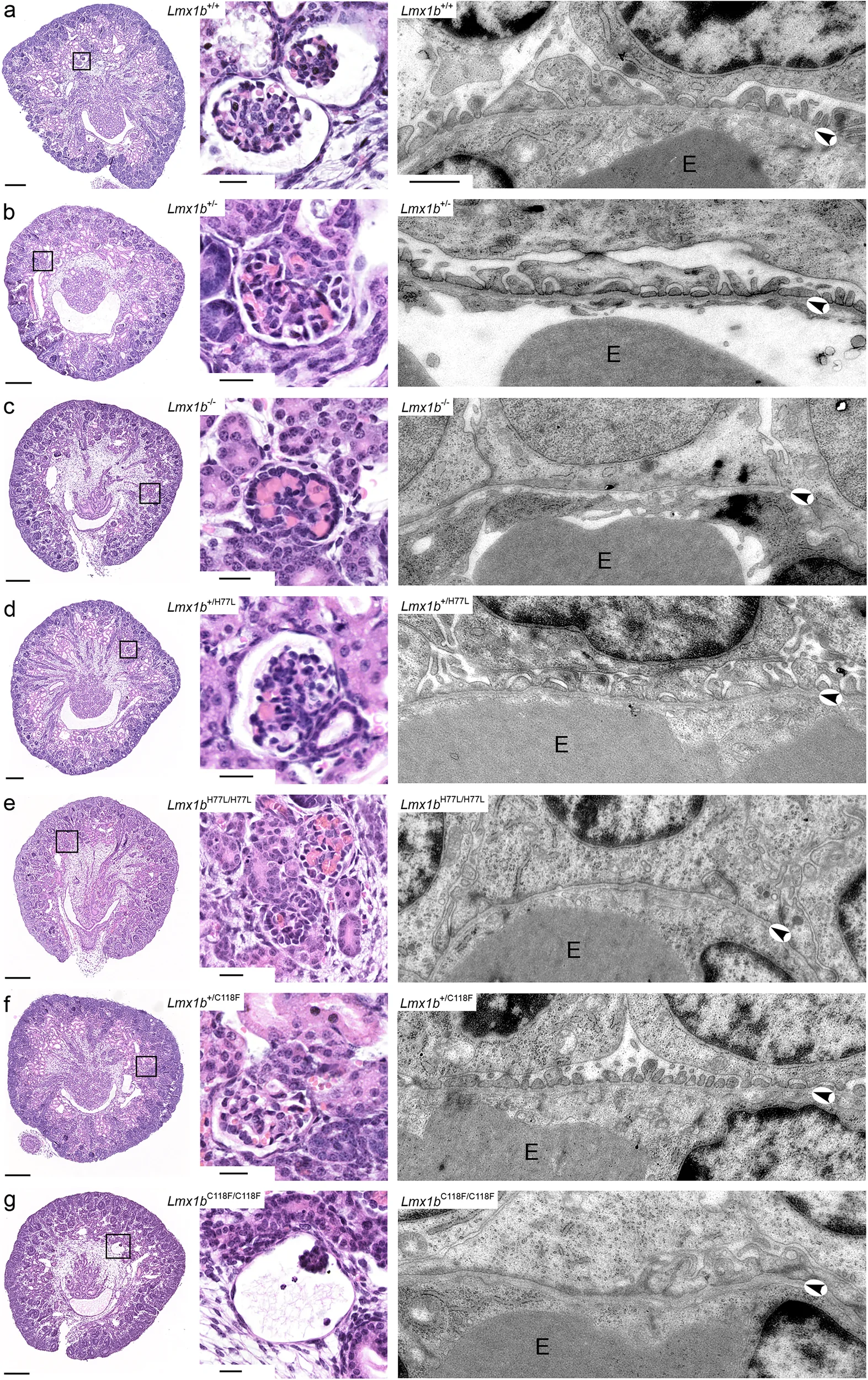
Renal phenotype of *Lmx1b*^H77L^ and *Lmx1b*^C118F^ knock-in mice. Mice were sacrificed on embryonic day 18.5, glomeruli shown are located at the cortico-medullary border. At least four mice were analyzed for each genotype. Already light microscopic pictures of whole kidneys (left column) and representative glomeruli (middle column) demonstrate changes in *Lmx1b*^H77L/H77L^ (**e**) and *Lmx1b*^C118F/C118F^ knock-in mice (**g**), regular *Lmx1b*^-/-^ knock-out mice served as a comparison (**c**). Ultrastructurally, the alteration of the glomerular filtration barrier (right column) can be appreciated by the disappearance of foot processes in *Lmx1b*^H77L/H77L^ and *Lmx1b*^C118F/C118F^ knock-in mice as well as in *Lmx1b*^-/-^ knock-out mice. No changes in comparison to *Lmx1b*^+/+^ wild-type mice (**a**) were found in *Lmx1b*^+/-^ knock-out mice (**b**), *Lmx1b*^+/H77L^ (**d**) and *Lmx1b*^+/C118F^ (**f**) knock-in mice. E, erythrocytes in glomerular capillaries; arrow heads point to the glomerular basement membrane. Scale bars, 200 μm (left column), 20 μm (middle column), 1 μm (right column).

Since the kidney symptoms determine the prognosis of the patients, we next turned to the characterization of the renal phenotype. Malformed glomeruli were already detected on H&E-stained kidney sections of both homozygous knock-in mice (Fig. 2e, g), but no abnormalities were seen in the kidneys of heterozygous knock-in mice (Fig. 2d, f). The light microscopic findings were reflected by ultrastructural defects in the sense that the number of foot processes was markedly reduced in the homozygous and not in the heterozygous knock-in mice (Fig. 2d–g). Therefore the situation in heterozygous and homozygous *Lmx1b* knock-in mice is equivalent to the situation in heterozygous and homozygous *Lmx1b* knock-out mice where a renal phenotype was only observed in *Lmx1b*^-/-^ animals [(Fig. 2b, c) and previous publications^5,6^].

### The LMX1B^H77L^ and LMX1B^C118F^ mutant proteins are unstable

The fact that the phenotypes of the two *Lmx1b* knock-in lines were indistinguishable from the phenotype of *Lmx1b*^-/-^ mice made us wonder whether the mutant proteins of the two knock-in alleles were still detectable. If their levels had dropped so drastically that they virtually mimicked a knock-out, this would explain the identical phenotypes. When we stained kidney sections of the *Lmx1b* knock-in mice with the polyclonal anti-Lmx1b antibody BMO8 we detected Lmx1b only in the podocytes of wild-type and heterozygous, but not in those of homozygous knock-in mouse kidneys (Supplementary Fig. 4). These results suggested that the phenotype we observed in the knock-in mice is due to a reduced stability of the mutant proteins. The trivial explanation that the mutant proteins were retained in the cytoplasm and did not reach the nucleus was ruled out by examining their location in a differentiated podocyte cell line where staining for the wild-type LMX1B, the LMX1B^H77L^ and the LMX1B^C118F^ proteins demonstrated a strong nuclear signal in all three cases (Supplementary Fig. 5).

**Fig. 3:**
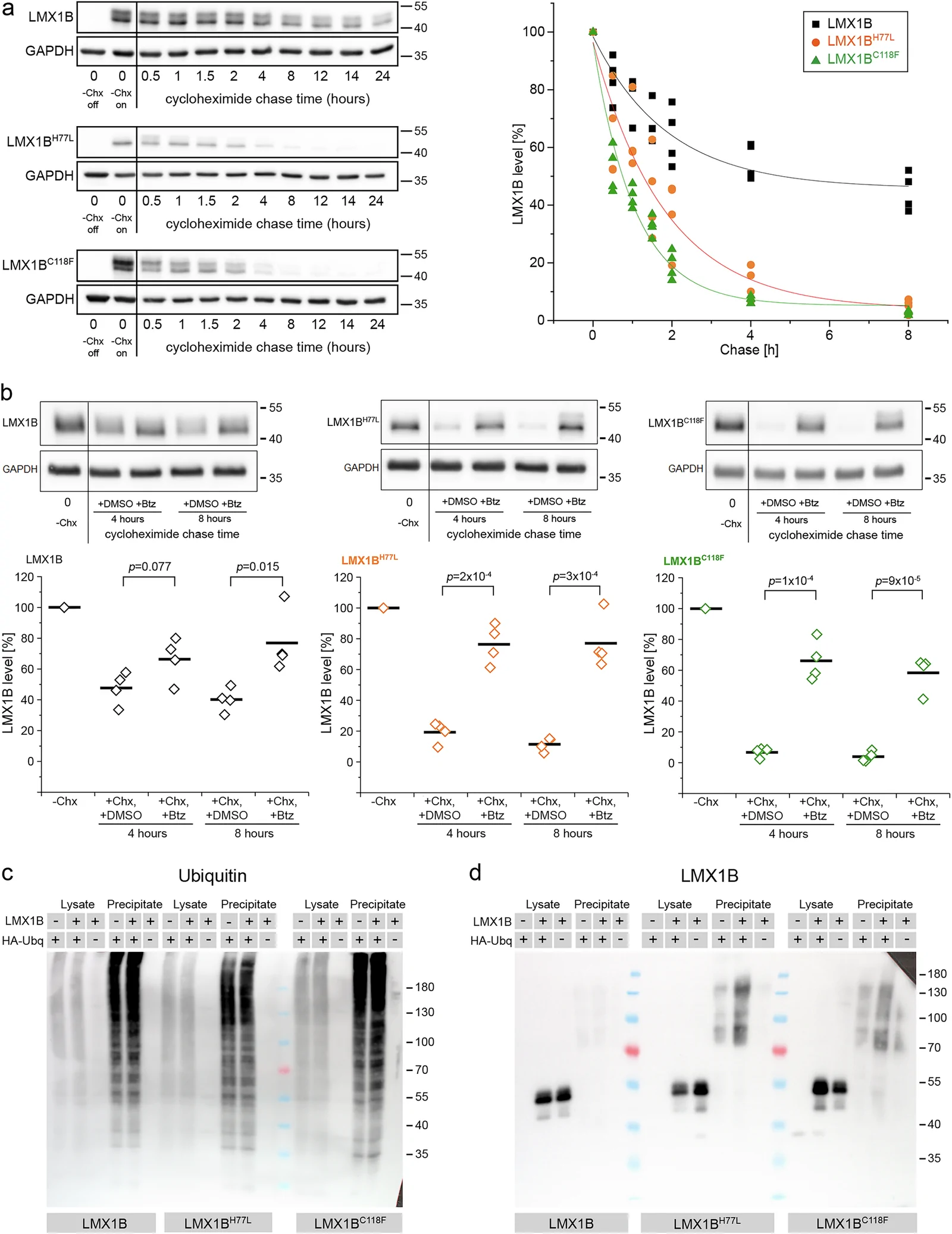
Stability of wild-type LMX1B, LMX1B^H77L^ and LMX1B^C118F^ proteins. Stably transfected HeLa cells inducibly producing human wild-type LMX1B, LMX1B^H77L^ and LMX1B^C118F^ proteins were used in the experiments. **a** Cells were harvested at the indicated time points and 40 μg of total protein were subjected to Western blot analysis, the numbers on the right of each blot give the molecular mass in kDa. Glyceraldehyde-3-phosphate dehydrogenase (GAPDH) was used as a loading control. Six days after initiating the induction of LMX1B (on), cycloheximide (Chx) was added into the culture medium to block protein synthesis (*t* = 0 hours). Cells in which LMX1B synthesis was not induced (off) are shown as a negative control. The results of 4 independent experiments are presented. **b** The experiment was carried out as described in (**a**), only that bortezomib (Btz), a pan-proteasomal inhibitor, was added at a final concentration of 10 μM together with cycloheximide which markedly prolonged the half-life of the mutant LMX1B proteins but had only a rather mild effect on the half-life of the wild-type protein. Control cells were treated with cycloheximide and DMSO as the solvent for bortezomib. Shown are the results of four independent experiments, the horizontal lines represent the mean values. **c**, **d** Four days after initiating the synthesis of the LMX1B, LMX1B^H77L^ and LMX1B^C118F^ proteins, cells were transiently transfected with an expression plasmid encoding a fusion protein of 7 ubiquitin units and a HA-tag at the NH_2_-terminus (HA-Ubq). Another two days later cells were lysed and lysates containing 400 μg of total protein immunoprecipitated with an antibody against the HA-tag. The same lysates (40 μg of total protein) and aliquots corresponding to 400 μg of immunoprecipitated proteins were Western blotted and incubated with antibodies against the HA-tag and against LMX1B. A representative result from 3 independent experiments is shown. Detection with an anti-HA antibody shows the smear resulting from the multitude of ubiquitinylated proteins in the immunoprecipitates (**c**), detection with an anti-LMX1B antibody shows the comparable expression levels of all three LMX1B proteins (cf. lysates) (**d**). A shift to higher molecular masses in the presence of the HA-tagged ubiquitin chains was observed only for the two mutant, but not the wild-type, LMX1B proteins (cf. precipitates). The faint smear in the off state probably results from the leaky expression of LMX1B even in the presence of doxycycline. Numbers on the right of each blot give the molecular mass in kDa. Statistical significance in panels **(a)** and **(b)** was determined by a two-sided unpaired Student’s *t* test. Source data are provided as a Source Data file.

In order to directly examine the hypothesis that the mutant proteins are unstable, we established stably transfected HeLa cells which synthesized human wild-type LMX1B, LMX1B^H77L^ and LMX1B^C118F^ proteins in a doxycycline-inducible fashion. After induction, cycloheximide was added to block new protein synthesis and total cell lysates were prepared over a 24 h time period. It was obvious that the two mutants, with 1.4 h for LMX1B^H77L^ and 0.8 h for LMX1B^C118F^, had a much shorter half-life than the wild-type protein with 4.8 h (Fig. 3a). The instability of proteins can be due to an increased degradation by the proteasome or autophagosome. We therefore again blocked protein synthesis with cycloheximide and at the same time added the pan-proteasomal inhibitor bortezomib^17^. The concomitant incubation of the cells with bortezomib led to a pronounced increase in the protein levels of the mutated proteins at the two time points investigated (Fig. 3b), indicating that they were subject to increased proteasomal degradation. This assumption was corroborated by experiments in which an alternative proteasome inhibitor was employed, MG132^18^, where again the levels of LMX1B^H77L^ and LMX1B^C118F^ were drastically higher in the presence of MG132 (Supplementary Fig. 6a). In contrast, the inhibition of vesicular proton pumps, which are necessary for the acidification of lysosomal compartments such as the autophagosome, with bafilomycin^19^ had no effect on the protein levels of the mutant proteins (Supplementary Fig. 6b). Further evidence for the proteasomal degradation of LMX1B^H77L^ and LMX1B^C118F^ was collected by investigating the ubiquitinylation of wild-type and mutant LMX1B proteins. Ubiquitinylation of proteins marks them for degradation by the proteasome^20^ and therefore is a signature of this pathway. The same cell lines used for the inhibitor experiments were transiently transfected with an expression plasmid encoding HA-tagged ubiquitin. Ubiquitinylated proteins were immunoprecipitated with an anti-HA-epitope antibody, and the precipitated proteins analyzed by Western blot with antibodies against the HA-epitope and against LMX1B. As expected from the ubiquitinylation of many endogenous proteins, a smear was observed with the anti-HA-epitope antibody (Fig. 3c). In addition, we detected higher molecular mass forms of the LMX1B mutant proteins but not of wild-type LMX1B, indicating that they are ubiquitinylated (Fig. 3d).

Patients with nail-patella syndrome typically carry mutations in the regions of *LMX1B* encoding the two LIM domains and the DNA-binding homeodomain^4^. Independent of the nature of the mutation, nail-patella syndrome presents as an autosomal-dominant disease. Therefore, it is puzzling that our *Lmx1b*^H77L^ and *Lmx1b*^C118F^ knock-in mouse lines develop a phenotype in a recessive fashion, whereas the phenotype in the ENU-induced *Lmx1b*^V265D^ mouse line, which suffers from a mutation in the homeodomain, has been reported to be inherited dominantly^21^. In order to find out why the LMX1B^V265D^ protein behaves differently from our LIM domain mutants, we performed similar experiments as those described above for the LMX1B^H77L^ and LMX1B^C118F^ mutant proteins. Remarkably the LMX1B^V265D^ protein was more stable than the wild-type LMX1B protein, its half-life was determined as 8.0 h (Supplementary Fig. 7a) compared to the 4.8 h of wild-type LMX1B (Fig. 3a). Bortezomib prolonged the half-life of LMX1B^V265D^, but the effect was modest (Supplementary Fig. 7b), and no effect was seen with bafilomycin (Supplementary Fig. 7c), similar to what we had observed for the wild-type protein (Fig. 3b and Supplementary Fig. 6b). The increased half-life of LMX1B^V265D^ may contribute to its dominant mode of action but we wondered whether other biochemical characteristics had changed. Therefore, we determined the surface potential of the homeodomain in the wild-type LMX1B and the LMX1B^V265D^ proteins. Homeodomains consist of three helices of which one inserts into the major groove of DNA. It is obvious that residue 265 is part of the DNA-binding helix and that the substitution of valine at this position by aspartate changes the surface potential to a more negative value by at least 3 eV (Supplementary Fig. 7d). This in turn would lead to a higher repulsion from the phosphate residues in the DNA, which explains the decreased binding of the mutant homeodomain observed before^21^.

### Treatment with bortezomib reduces proteinuria in compound heterozygous Lmx1b knock-out and knock-in mice

**Fig. 4:**
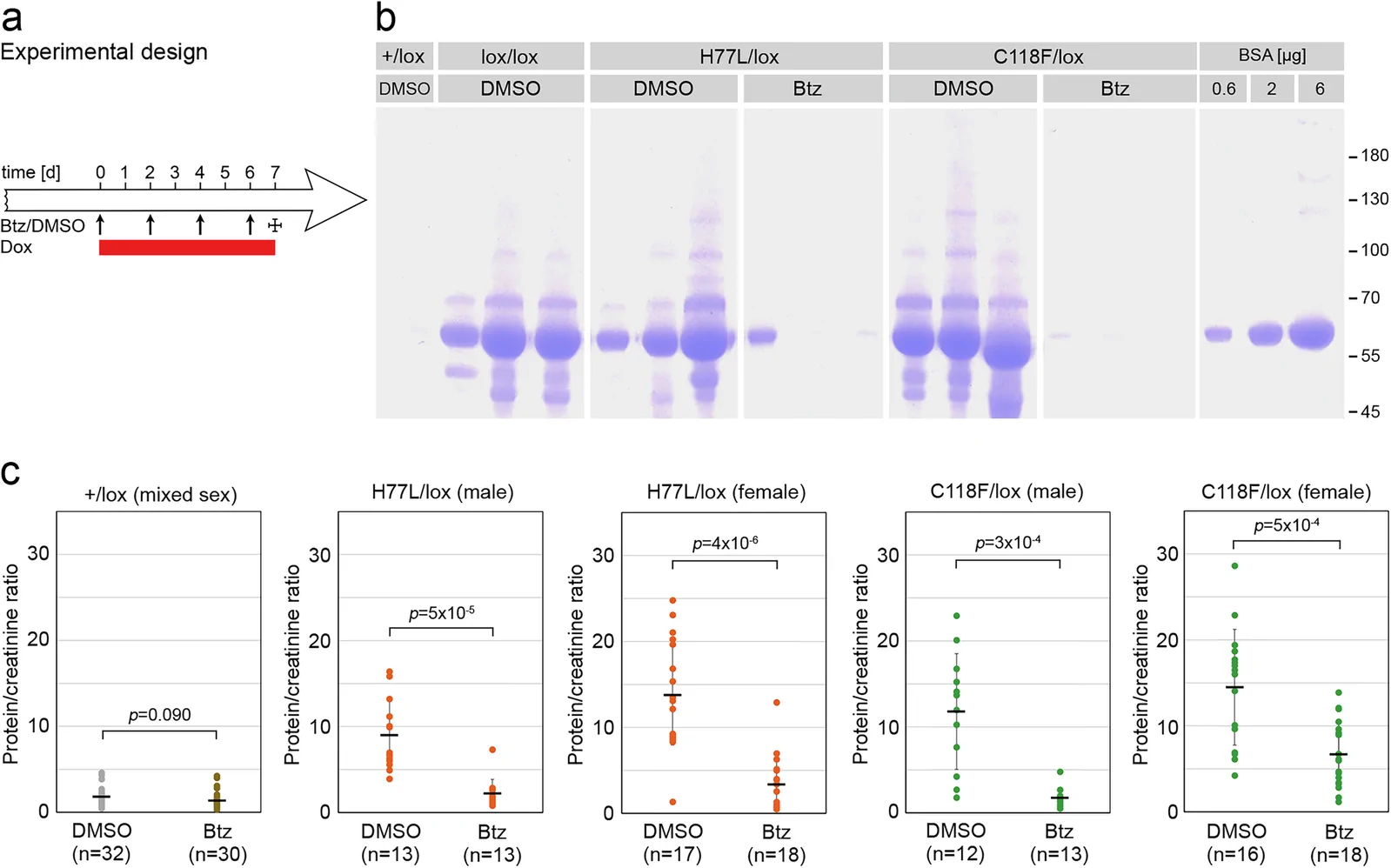
Bortezomib alleviates proteinuria in *Lmx1b*^H77L/lox^ and *Lmx1b*^C118F/lox^ mice. Mice between 7–10 weeks of age were entered into the experiment. In addition to the mutated *Lmx1b* alleles, the mice also contained expression cassettes for the doxycycline-inducible, podocyte-specific expression of Cre recombinase (*Lmx1b*^H77L/lox^ / *NPHS2*::rtTA / tetO::Cre mice, and *Lmx1b*^C118F/lox^ / *NPHS2*::rtTA / tetO::Cre mice). **a** Time course of the experimental strategy. Inactivation of the floxed *Lmx1b* allele was achieved by adding 2 mg/ml of doxycycline to the drinking water for a period of seven days. Bortezomib (Btz) was injected intraperitoneally every other day beginning with the administration of doxycycline, DMSO as the solvent for bortezomib served as a negative control. Spot urine was collected on day 7 before the mice were perfusion-fixed. **b** 0.4 μl of urine from male mice were loaded on a protein gel together with 0.6, 2 and 6 μg of bovine serum albumin (BSA). As published previously, *Lmx1b*^+/lox^ / *NPHS2*::rtTA / tetO::Cre mice (+/lox) did not develop proteinuria and *Lmx1b*^lox/lox^ / *NPHS2*::rtTA / tetO::Cre mice (lox/lox) developed strong proteinuria. Bortezomib markedly reduced proteinuria in both *Lmx1b*^H77L/lox^ / *NPHS2*::rtTA / tetO::Cre (H77L/lox) and *Lmx1b*^C118F/lox^ / *NPHS2*::rtTA / tetO::Cre (C118F/lox) mice. The numbers on the right of each blot give the molecular mass in kDa. **c** The administration of bortezomib to *Lmx1b*^+/lox^ / *NPHS2*::rtTA / tetO::Cre mice does not cause proteinuria but alleviates proteinuria in both male and female *Lmx1b*^H77L/lox^ / *NPHS2*::rtTA / tetO::Cre and *Lmx1b*^C118F/lox^ / *NPHS2*::rtTA / tetO::Cre mice. Each dot represents the protein/creatinine ratio of an individual mouse, the horizontal and vertical lines indicate the mean values and standard deviations, respectively. Statistical significance was determined by a two-sided unpaired Student’s *t* test. Source data are provided as a Source Data file.

**Fig. 5:**
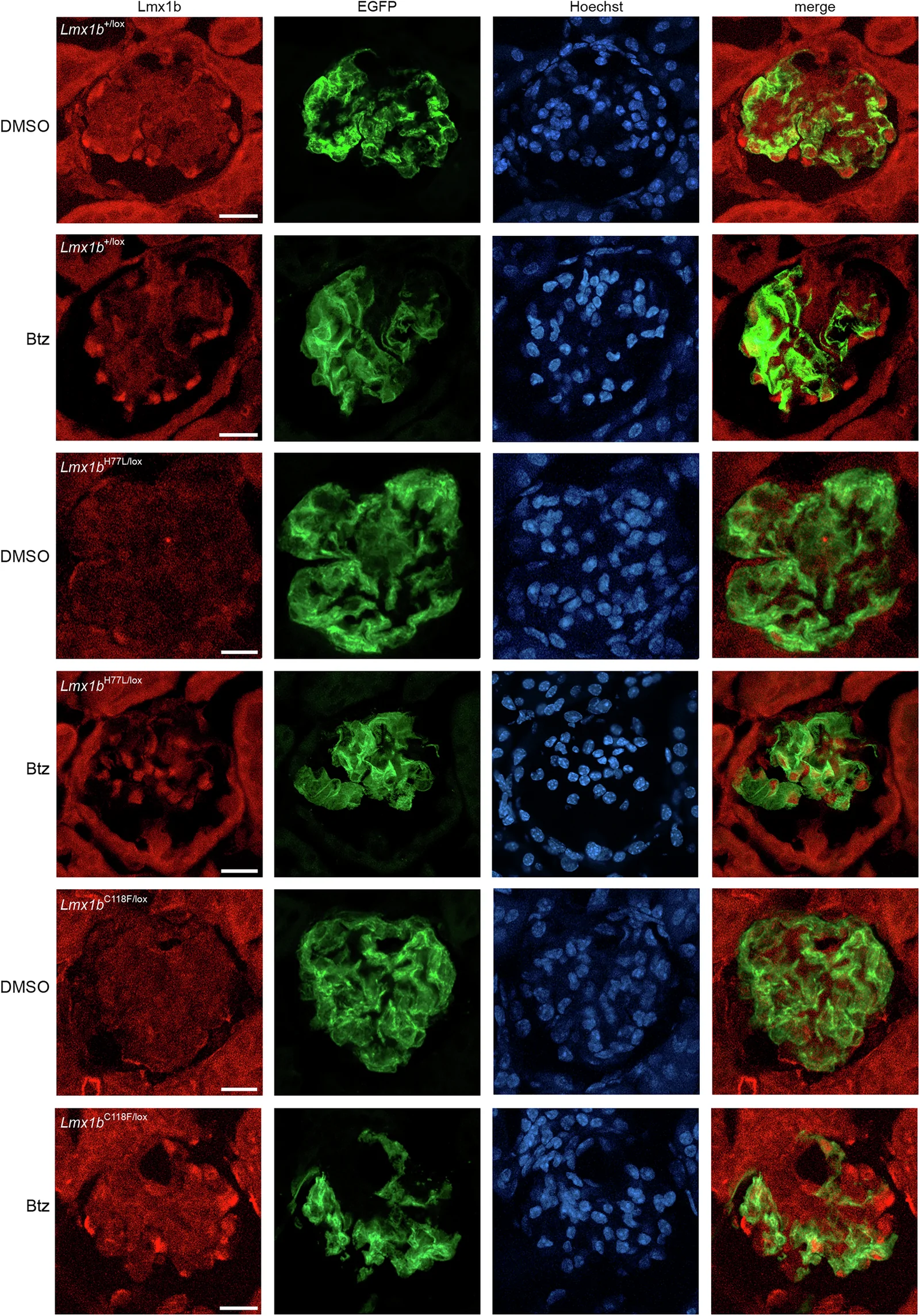
Bortezomib stabilizes the Lmx1b^H77L^ and Lmx1b^C118F^ proteins in vivo. Kidney sections of mice between 7–10 weeks of age are shown. In addition to the mutated *Lmx1b* alleles, the mice contained expression cassettes (i) for the doxycycline-inducible, podocyte-specific expression of Cre recombinase and (ii) for the Cre-mediated expression of membrane-targeted EGFP (*Lmx1b*^H77L/lox^ / *NPHS2*::rtTA / tetO::Cre / mTmG and *Lmx1b*^C118F/lox^ / *NPHS2*::rtTA / tetO::Cre / mTmG mice). Inactivation of the floxed *Lmx1b* allele and expression of EGFP was achieved by adding 2 mg/ml of doxycycline to the drinking water for a period of seven days. Bortezomib (Btz) was injected intraperitoneally every other day beginning with the administration of doxycycline, equivalent volumes of DMSO were injected as a negative control. The induction of Cre in podocytes was detected through EGFP fluorescence, the presence of Lmx1b was detected with an anti-Lmx1b antibody and an Alexa Fluor 633-conjugated secondary antibody. It can be seen that Lmx1b is present in mice treated with bortezomib but not in those which received DMSO. Compound heterozygous mice containing one wild-type allele of *Lmx1b* served as a positive control for the presence of Lmx1b. Co-staining with the dye Hoechst 33258 (Hoechst) demonstrated the presence of wild-type and mutant Lmx1b proteins in the nuclei of podocytes. Five mice were analyzed for each genotype and treatment, a representative result in each case is shown. Scale bar, 20 μm.

**Fig. 6:**
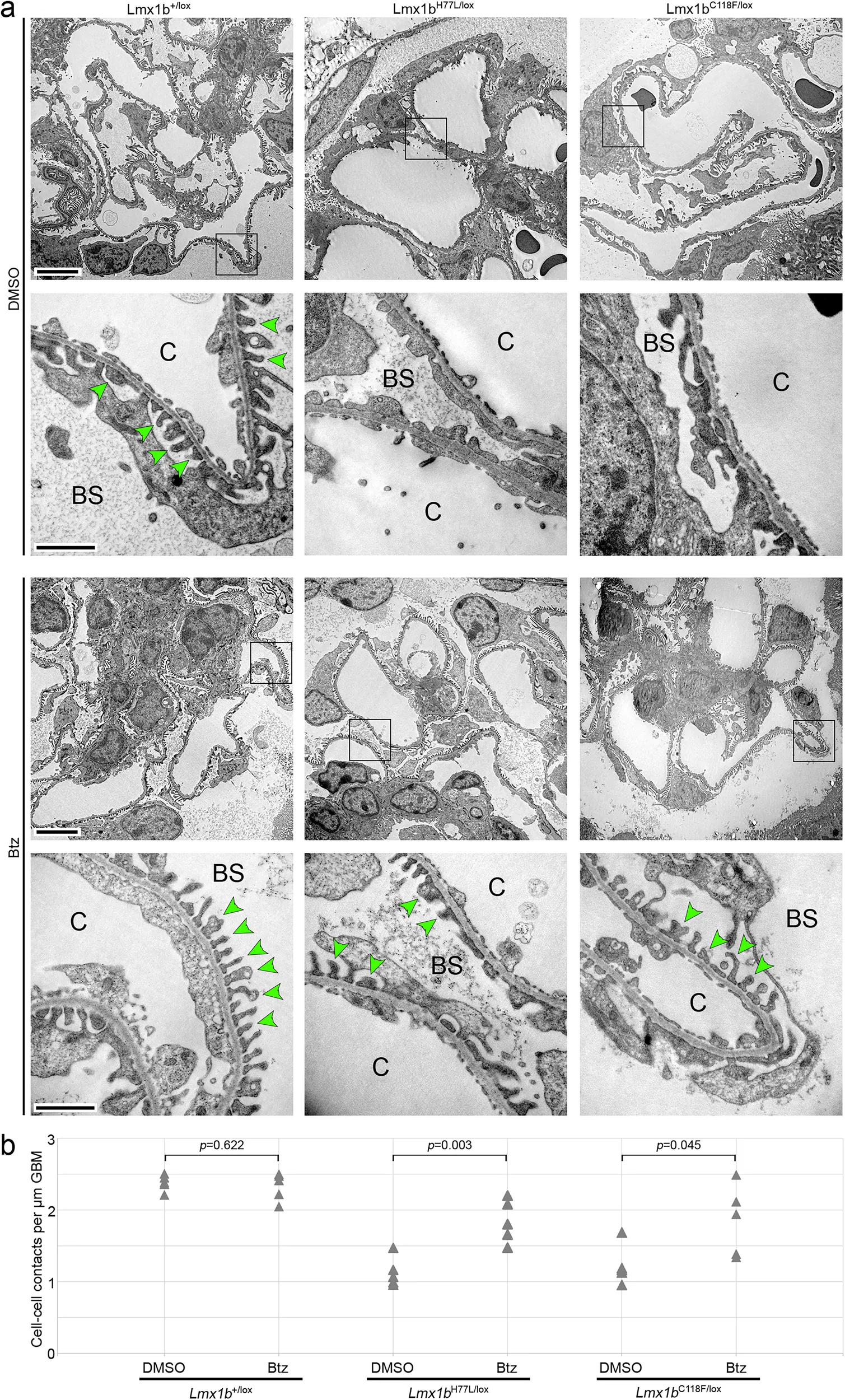
Treatment with bortezomib maintains the glomerular filtration barrier. Mice between 7–10 weeks of age were characterized. In addition to the mutated *Lmx1b* alleles, the mice also contained expression cassettes for the doxycycline-inducible, podocyte-specific expression of Cre recombinase (*Lmx1b*^H77L/lox^ / *NPHS2*::rtTA / tetO::Cre and *Lmx1b*^C118F/lox^ / *NPHS2*::rtTA / tetO::Cre mice). Inactivation of the floxed *Lmx1b* allele was achieved by adding 2 mg/ml of doxycycline to the drinking water for a period of 7 days. Bortezomib (Btz) was injected intraperitoneally every other day beginning with the administration of doxycycline, DMSO as the solvent for bortezomib served as a negative control. **a** Transmission electron micrographs demonstrate the drastically reduced number of foot processes (arrow heads) after 7 days of Cre induction (top panels), which was prevented to a large degree by the administration of bortezomib (bottom panels). *Lmx1b*^+/lox^ mice served as a negative control and demonstrate that bortezomib did not damage podocytes. C, capillary lumen; BS, Bowman’s space. **b** Quantitation of the podocytic cell-cell contacts demonstrates the beneficial effect of bortezomib. Since in the case of the DMSO-treated mice, many cell-cell contacts did not represent typical filtration slits with a slit diaphragm, the degree of podocyte damage in the DMSO-treated mice and therefore the beneficial effect of bortezomib is underestimated. *n* = 5 mice in each group. Statistical significance was determined by a two-sided unpaired Student’s *t* test. Scale bars, 5 μm (overview), 1 μm (large magnification). Source data are provided as a Source Data file.

The results presented above provide strong evidence that the phenotype observed in the homozygous *Lmx1b*^H77L/H77L^ and *Lmx1b*^C118F/C118F^ mice is caused by the instability of the respective mutant proteins. At the same time this scenario opens up the opportunity for a therapeutic intervention because bortezomib is employed as a drug against multiple myeloma^22,23^. If the mutant Lmx1b proteins were still functional and could be stabilized through the action of bortezomib, we expected to see a beneficial effect in the knock-in mice. In order to find out whether bortezomib may also be useful in the setting of nail-patella syndrome, we established compound heterozygous *Lmx1b*^H77L/lox^ and *Lmx1b*^C118F/lox^ mice, which in addition contained expression cassettes for the inducible podocyte-specific synthesis of Cre recombinase (*NPHS2*::rtTA / tetO::Cre mice). We showed previously that the podocyte-specific inactivation of *Lmx1b* upon the doxycycline-induced expression of Cre recombinase in *Lmx1b*^lox/lox^ mice leads to strong proteinuria in adult mice after seven days of induction^8^. Both compound heterozygous mouse lines (*Lmx1b*^H77L/lox^ / *NPHS2*::rtTA / tetO::Cre mice, and *Lmx1b*^C118F/lox^ / *NPHS2*::rtTA / tetO::Cre mice) developed no phenotype in the absence of Cre recombinase, but after the doxycycline-mediated recombination of the floxed allele we observed marked proteinuria (Fig. 4b), which again demonstrates that the mutant alleles behave like the null allele. The administration of bortezomib every other day did not show renal toxicity as judged by proteinuria (Fig. 4c) and electron microscopy (see below, Fig. 6). When we administered bortezomib according to the same regimen at the time of induction of Cre recombinase we observed a pronounced reduction of proteinuria after seven days for either knock-in allele and for both female and male mice (Fig. 4b, c). In order to explain this impressive therapeutic effect, the kidneys of all experimental groups were stained for Lmx1b and characterized by electron microscopy. Compared to DMSO-treated mice, bortezomib-treated *Lmx1b*^H77L/lox^ and *Lmx1b*^C118F/lox^ mice showed an increased number of Lmx1b-positive podocytes (Fig. 5). Consistent with the beneficial effect of bortezomib on proteinuria, more regularly shaped foot processes and more cell-cell contacts were determined for podocytes (Fig. 6). In the latter case we decided to err to our disadvantage and did not try to differentiate between true filtration slits and other kinds of cell-cell contacts, otherwise the beneficial effect of bortezomib would probably have come out even more pronounced.

**Fig. 7:**
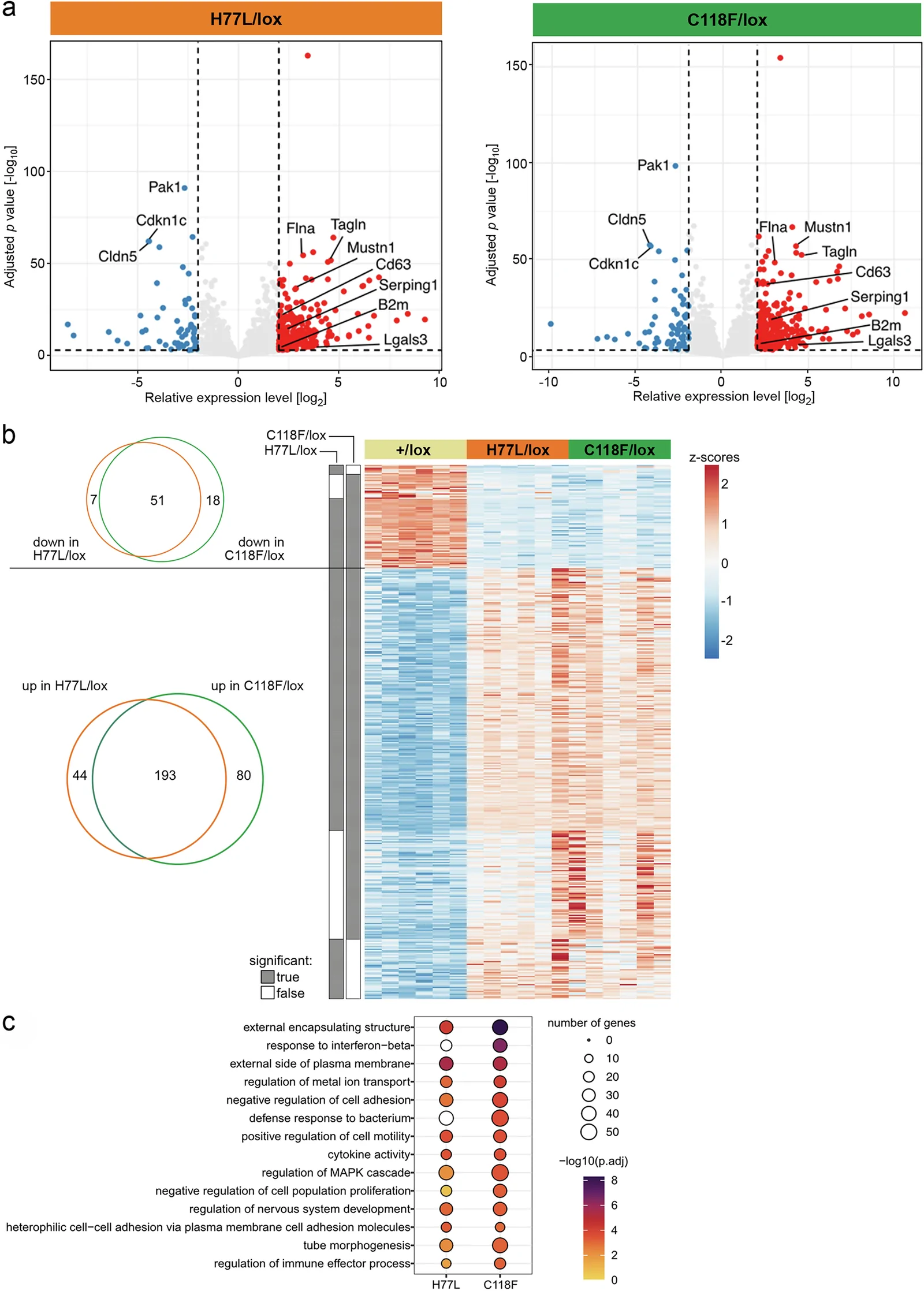
Differential gene expression in podocytes of *Lmx1b*^H77L/lox^ and *Lmx1b*^C118F/lox^ knock-in mice. *Lmx1b*^+/lox^, *Lmx1b*^H77L/lox^ and *Lmx1b*^C118F/lox^ mice with a podocyte-specific, doxycycline-inducible expression cassette for Cre recombinase were used in the experiments, furthermore the mice contained an expression cassette for the Cre-mediated expression of membrane-targeted EGFP (*Lmx1b*^+/lox^ / *NPHS2*::rtTA / tetO::Cre / mTmG mice; *Lmx1b*^H77L/lox^ / *NPHS2*::rtTA / tetO::Cre / mTmG mice; *Lmx1b*^C118F/lox^ / *NPHS2*::rtTA / tetO::Cre / mTmG mice). Mice were between 7–10 weeks of age. Induction of Cre recombinase was started on day 0 by the administration of doxycycline in the drinking water together with the intraperitoneal injection of DMSO (solvent for bortezomib) every other day. On day 7, green fluorescent podocytes were collected by FACS, and total RNA from the freshly isolated podocytes was sequenced. **a** A volcano plot illustrates the genes with a significant difference in expression. Shown are the log_2_fold changes compared to the podocytes of DMSO-treated *Lmx1b*^+/lox^ / *NPHS2*::rtTA / tetO::Cre / mTmG mice with a minimum change of log_2_ > 2 and a false discovery rate (FDR) of 0.001. *p-*values and log_2_fold changes were calculated using DESeq2, which uses a negative binominal GLM (generalized linear model) and Wald statistics, and were adjusted for multiple testing using the false discovery rate (FDR). The genes whose expression was further analyzed by immunofluorescence and qPCR are highlighted (gene - protein pairs): *B2m*, β2-microglobulin; *Cd63* - Cd63 antigen; *Cdkn1c* - cyclin-dependent kinase inhibitor 1c, p57; *Cldn5* - claudin-5; *Flna* - filamin A; *Lgals3* - galectin-3; *Mustn1* - mustn1, musculoskeletal embryonic nuclear protein 1; *Pak1* - Pak1, p21-activated kinase 1; *Serping1* - serping1, complement component 1 inhibitor; *Tagln* - transgelin. **b** In the case of the inducible *Lmx1b*^H77L/lox^ mice, 58 genes were down- and 237 genes were up-regulated, in the case of the inducible *Lmx1b*^C118F/lox^ mice 69 genes were down- and 273 genes were up-regulated. Venn diagrams illustrate the overlap of the differentially regulated genes, the heatmap shows the *z* scores of rlog-normalized expression counts. The genotypes in which the genes are significantly regulated can be seen on the left side of the heatmap. **c** Metascape gene ontology (GO) enrichment analysis of genes with significantly different expression levels. The summary terms of similarity-grouped GO terms are depicted. The size of the dots represents the number of genes, the color of the dots indicates the adjusted *p-*value with white being below the cutoff of 0.01. Statistical significance was assessed using a one-sided cumulative hypergeometric test to evaluate over-representation, and *p-*values were adjusted for multiple testing using the false discovery rate (FDR). +/lox, *Lmx1b*^+/lox^ / *NPHS2*::rtTA / tetO::Cre / mTmG mice; H77L/lox and H77L, *Lmx1b*^H77L/lox^ / *NPHS2*::rtTA / tetO::Cre / mTmG mice; C118F/lox and C118F, *Lmx1b*^C118F/lox^ / *NPHS2*::rtTA / tetO::Cre / mTmG mice. *n* = 6 mice in each group.

Utilizing the inducible podocyte-specific *Lmx1b*^lox/lox^ knock-out mice (*Lmx1b*^lox/lox^ / *NPHS2*::rtTA / tetO::Cre mice) we presented evidence that genes encoding actin-associated proteins are regulated by Lmx1b^8^. This previous analysis was carried out with isolated glomeruli, and we therefore may have missed Lmx1b target genes because of the increased background noise due to the additional presence of mesangial and endothelial cells in glomerular preparations. The *Lmx1b*^H77L/lox^ and *Lmx1b*^C118F/lox^ mice not only allowed us to investigate whether the LIM-A and LIM-B domains of Lmx1b carry out gene-specific functions, but by mating them with the mT/mG mouse line^24^ we were also able to specifically isolate podocytes and thereby fine-tune our expression analysis. The comparison between the podocytes of DMSO-treated *Lmx1b*^+/lox^ and *Lmx1b*^H77L/lox^ mice (DMSO served as the solvent for bortezomib, see below) revealed that 58 genes were down- and 237 genes were up-regulated after seven days of Cre induction (cutoffs were set at log_2_fold change > 2 and *p* < 0.001) (Fig. 7a, b). In the case of the DMSO-treated *Lmx1b*^C118F/lox^ mice, 69 genes were down- and 273 genes were up-regulated after seven days of Cre induction in comparison to *Lmx1b*^+/lox^ mice. No significant changes were observed for 10,665 and 10,553 genes, respectively. Most of the down- and up-regulated genes overlapped between the two compound heterozygous mouse lines (Fig. 7b). Of the down-regulated genes, 51 overlapped, and of the upregulated genes, 193 overlapped between the two genotypes, meaning that the majority of regulated genes were shared between the two mouse lines (Fig. 7b). A gene ontology (GO) analysis also revealed many overlaps between the two compound heterozygous mouse lines such as genes that were connected to the extracellular matrix (‘external encapsulating structure’) and cell adhesion (e.g., ‘negative regulation of cell adhesion’, ‘heterophilic cell-cell adhesion via plasma membrane cell adhesion molecules’). However, the overlap was less pronounced for other gene families, such as the ‘response to interferon-beta’ (Fig. 7c).

**Fig. 8:**
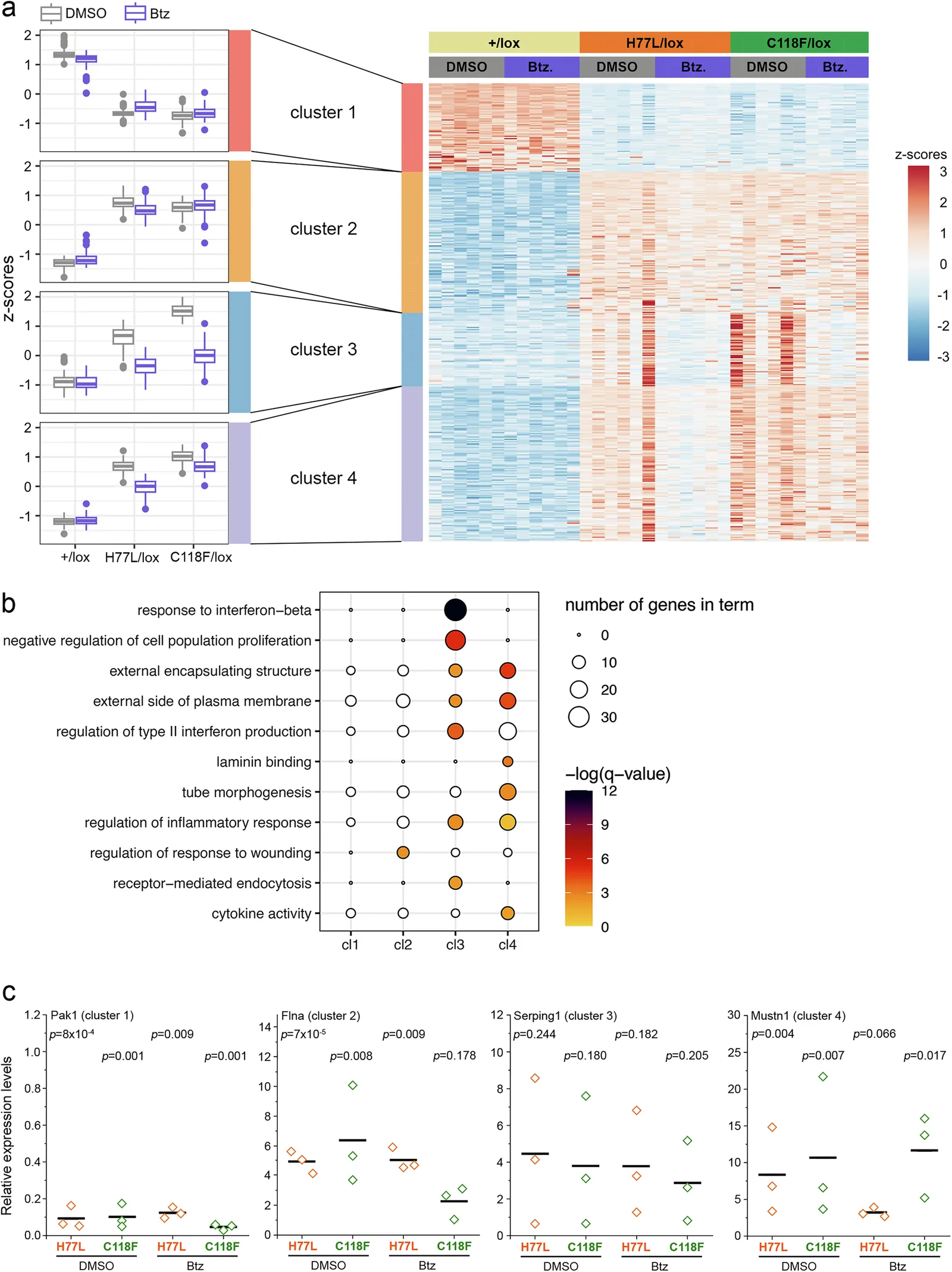
Treatment with bortezomib partially reverses the effect of mutations in the LIM domains. *Lmx1b*^+/lox^, *Lmx1b*^H77L/lox^ and *Lmx1b*^C118F/lox^ mice with a podocyte-specific, doxycycline-inducible expression cassette for Cre recombinase were used in the experiments, furthermore the mice contained an expression cassette for the Cre-mediated expression of membrane-targeted EGFP (*Lmx1b*^+/lox^ / *NPHS2*::rtTA / tetO::Cre / mTmG mice; *Lmx1b*^H77L/lox^ / *NPHS2*::rtTA / tetO::Cre / mTmG mice; *Lmx1b*^C118F/lox^ / *NPHS2*::rtTA / tetO::Cre / mTmG mice). Mice were between 7–10 weeks of age. Induction of Cre recombinase was started on day 0 by the administration of doxycycline in the drinking water together with the intraperitoneal injection of DMSO (solvent for bortezomib) or bortezomib every other day. On day 7, green fluorescent podocytes were collected by FACS, and total RNA from the freshly isolated podocytes was isolated. **a** K-means clustering of genes which are differentially regulated at a statistically significant level in either the inducible *Lmx1b*^H77/lox^ or *Lmx1b*^C118F/lox^ mice or both under DMSO treatment compared to the inducible *Lmx1b*^+/lox^ mice. An identical analysis was carried out for mice treated with bortezomib. The group median of the rlog-transformed gene expression counts was extracted and scaled to *z* scores. A heatmap shows the rlog-transformed gene expression counts for each sample divided into the corresponding clusters. Box plots on the left side depict the median *z* scores split up by the corresponding clusters. The first two clusters contain genes whose expression levels decreased (cluster 1) or increased (cluster 2) in both knock-in lines after Cre-mediated inactivation of the floxed *Lmx1b* allele, regardless of the administration of bortezomib. Cluster 3 contains genes whose expression levels rose in both knock-in lines after Cre-mediated inactivation of the floxed *Lmx1b* allele, but their increase was prevented in both lines by the administration of bortezomib. Cluster 4 contains genes whose expression levels rose in both knock-in lines after Cre-mediated inactivation of the floxed *Lmx1b* allele, but whose increase was prevented by bortezomib only in the *Lmx1b*^H77L/lox^ mouse line. Boxes represent the interquartile range (IQR), the line inside a box indicates the median of the dataset, and the whiskers extend from the edges of a box to the first and third quartiles, but no further than 1.5 times of the IQR. Any data points outside this range are plotted individually as dots. **b** Metascape gene ontology (GO) enrichment analysis of the gene clusters. The summary terms of similarity-grouped GO terms are depicted. The size of the dots represents the number of genes, the color of the dots indicates the adjusted *p-*value with white being below the cutoff of 0.01. *n* = 6 mice in each group, except for the *Lmx1b*^C118F/lox^ mice treated with bortezomib where *n* = 5. **c** RT-qPCR shows the downregulation of the *Pak1* gene in both knock-in lines after DMSO and bortezomib treatment, the upregulation of *Flna* after DMSO treatment in both lines and after bortezomib treatment in the *Lmx1b*^H77L/lox^ knock-in line, no significant changes for *Serping1*, and the lack of a significant increase of *Mustn1* after bortezomib treatment in the *Lmx1b*^H77L/lox^ knock-in line. Three mice were analyzed in each group. Statistical significance was determined by a two-sided unpaired Student’s *t* test. +/lox, *Lmx1b*^+/lox^ / *NPHS2*::rtTA / tetO::Cre / mTmG mice; H77L/lox and H77L, *Lmx1b*^H77L/lox^ / *NPHS2*::rtTA / tetO::Cre / mTmG mice; C118F/lox and C118F, *Lmx1b*^C118F/lox^ / *NPHS2*::rtTA / tetO::Cre / mTmG mice. Source data are provided as a Source Data file.

When bortezomib was administered to *Lmx1b*^+/lox^ / *NPHS2*::rtTA / tetO::Cre mice every other day, no obvious difference was detected to the DMSO-treated *Lmx1b*^+/lox^ / *NPHS2*::rtTA / tetO::Cre animals (Fig. 8a). This is consistent with the observation that *Lmx1b*^+/lox^ mice treated with bortezomib did not develop proteinuria (Fig. 4c) and *Lmx1b*^+/+^ mice treated with bortezomib did not show foot process effacement (Fig. 6). A bioinformatic analysis yielded four patterns after bortezomib treatment of *Lmx1b*^H77L/lox^ / *NPHS2*::rtTA / tetO::Cre and *Lmx1b*^C118F/lox^ / *NPHS2*::rtTA / tetO::Cre mice (Fig. 8a). In the first cluster of genes expression levels decreased in the podocytes of both knock-in lines independent of the fact whether bortezomib was administered or not. In the second cluster of genes, expression levels rose in the podocytes of both knock-in lines again independently of bortezomib treatment. In the third cluster, bortezomib treatment prevented the rise in expression levels in the podocytes of the respective genes in both knock-in lines. Finally, in the fourth cluster of genes, expression levels rose in the podocytes of both compound heterozygous mouse lines, but this increase was prevented only by bortezomib treatment in the *Lmx1b*^H77L/lox^ mouse line. The genes in this cluster may provide an answer to the question whether any genes are differentially regulated between the *Lmx1b*^H77L/lox^ and *Lmx1b*^C118F/lox^ mice, i.e., whether the LIM domains serve distinct functions in podocytes. A GO analysis of the clusters emphasized which gene families responded similarly to bortezomib treatment in both mouse lines (e.g., ‘response to interferon-beta’, ‘negative regulation of cell population proliferation’) and which gene families were regulated in a more LIM domain-specific manner (e.g., ‘external encapsulating structure’, ‘external side of plasma membrane’) (Fig. 8b). We verified the expression of exemplary mRNAs from all four clusters. PAK1 (also known as p21-activated kinase 1) is produced by podocytes of healthy kidneys^25,26^ where it is believed to regulate the actin cytoskeleton. It is strongly downregulated in the podocytes of *Lmx1b*^H77L/lox^ / *NPHS2*::rtTA / tetO::Cre and *Lmx1b*^C118F/lox^ / *NPHS2*::rtTA / tetO::Cre mice independently of the administration of DMSO and bortezomib, consistent with it belonging to cluster 1 (Fig. 8c). In previous publications filamins, which constitute a family of actin-binding proteins, have been found to be upregulated in damaged podocytes^27,28^. Filamin A belongs to cluster 2, where mRNA levels should increase in both knock-in mouse lines after DMSO and bortezomib treatment. However, although the mRNA levels of filamin A rose significantly in both DMSO-treated knock-in lines and the bortezomib-treated *Lmx1b*^H77L/lox^ / *NPHS2*::rtTA / tetO::Cre mice, the increase did not reach statistical significance in bortezomib-treated *Lmx1b*^C118F/lox^ / *NPHS2*::rtTA / tetO::Cre mice (Fig. 8c). The *Serping1* gene encodes the complement component 1 inhibitor and there is some evidence that it is activated in podocytes during IgA nephropathy^29^, we investigated its mRNA levels as a representative of cluster 3. Since we did not observe a statistically significant increase in both DMSO-treated knock-in lines, the difference observed by RNA sequencing could not be reproduced (Fig. 8c). The *Mustn1* gene codes for musculoskeletal embryonic nuclear protein 1, nothing is known about its function in the kidney. It belongs to cluster 4, where an upregulation of the respective mRNAs is observed in both DMSO-treated knock-in mouse lines, but no increase after bortezomib treatment in the *Lmx1b*^H77L/lox^ / *NPHS2*::rtTA / tetO::Cre line. This pattern was confirmed by qPCR (Fig. 8c).

**Fig. 9:**
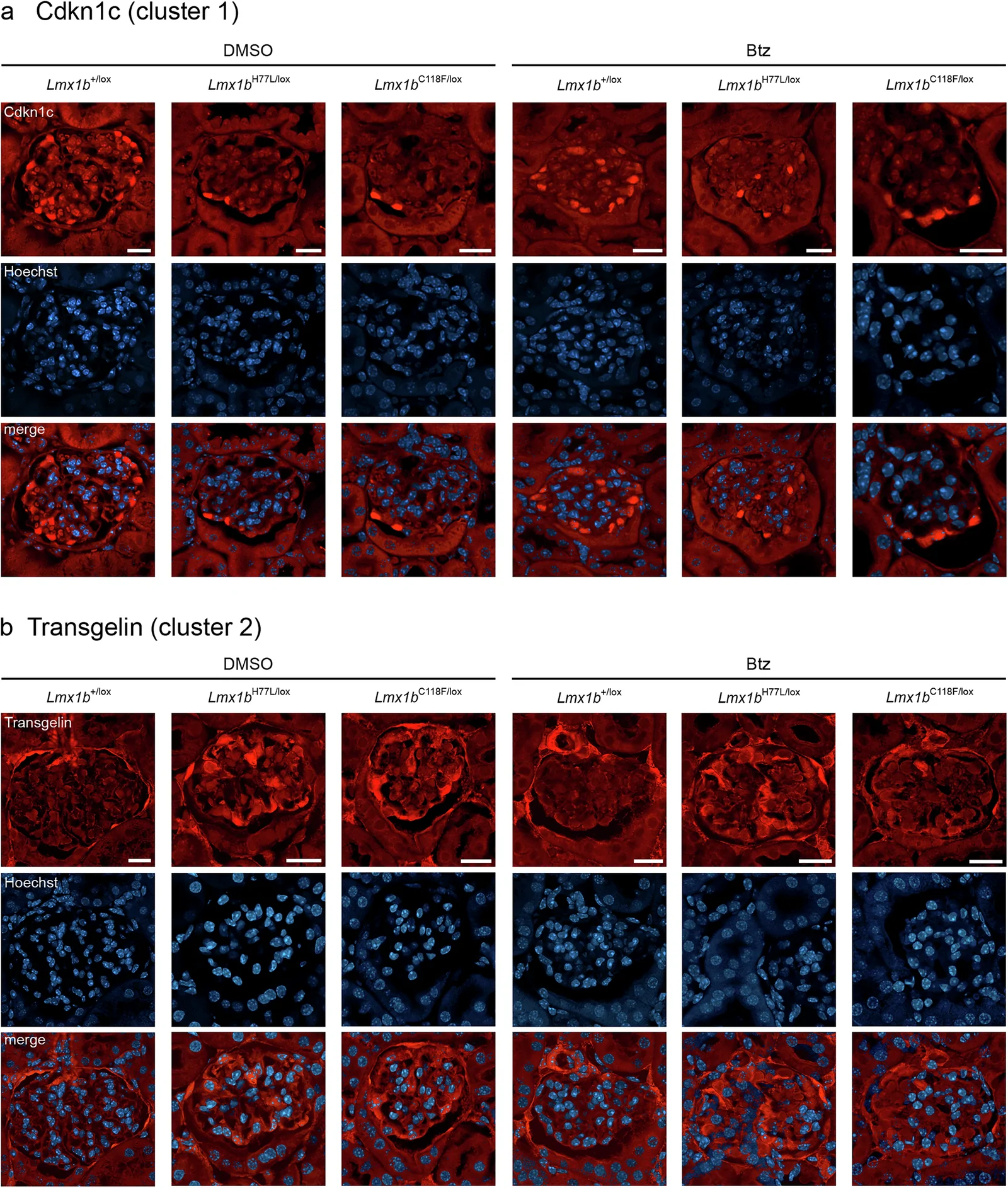
Protein expression pattern after treatment of *Lmx1b*^H77L/lox^ and *Lmx1b*^C118F/lox^ knock-in mice with DMSO and bortezomib. *Lmx1b*^+/lox^, *Lmx1b*^H77L/lox^ and *Lmx1b*^C118F/lox^ mice with a podocyte-specific, doxycycline-inducible expression cassette for Cre recombinase (*Lmx1b*^+/lox^ / *NPHS2*::rtTA / tetO::Cre mice; *Lmx1b*^H77L/lox^ / *NPHS2*::rtTA / tetO::Cre mice; *Lmx1b*^C118F/lox^ / *NPHS2*::rtTA / tetO::Cre mice) were analyzed. Mice were between 7–10 weeks of age. Induction of Cre recombinase was started on day 0 by the administration of doxycycline in the drinking water together with the intraperitoneal injection of DMSO (solvent for bortezomib) or bortezomib every other day. On day 7, mice were perfusion-fixed, and kidney sections were stained with the respective antibodies. Nuclei were counter-stained with Hoechst 33258 (Hoechst). Three mice were analyzed for each genotype and treatment, a representative result in each case is shown. **a** In the case of the cell cycle inhibitor Cdkn1c, a markedly lower number of positive podocyte nuclei can be seen in both knock-in lines independently of bortezomib treatment. **b** The actin-associated protein transgelin is absent in DMSO- and bortezomib-treated *Lmx1b*^+/lox^ mice but is expressed in the podocytes of both knock-in lines independently of bortezomib treatment. Scale bars, 20 μm.

**Fig. 10:**
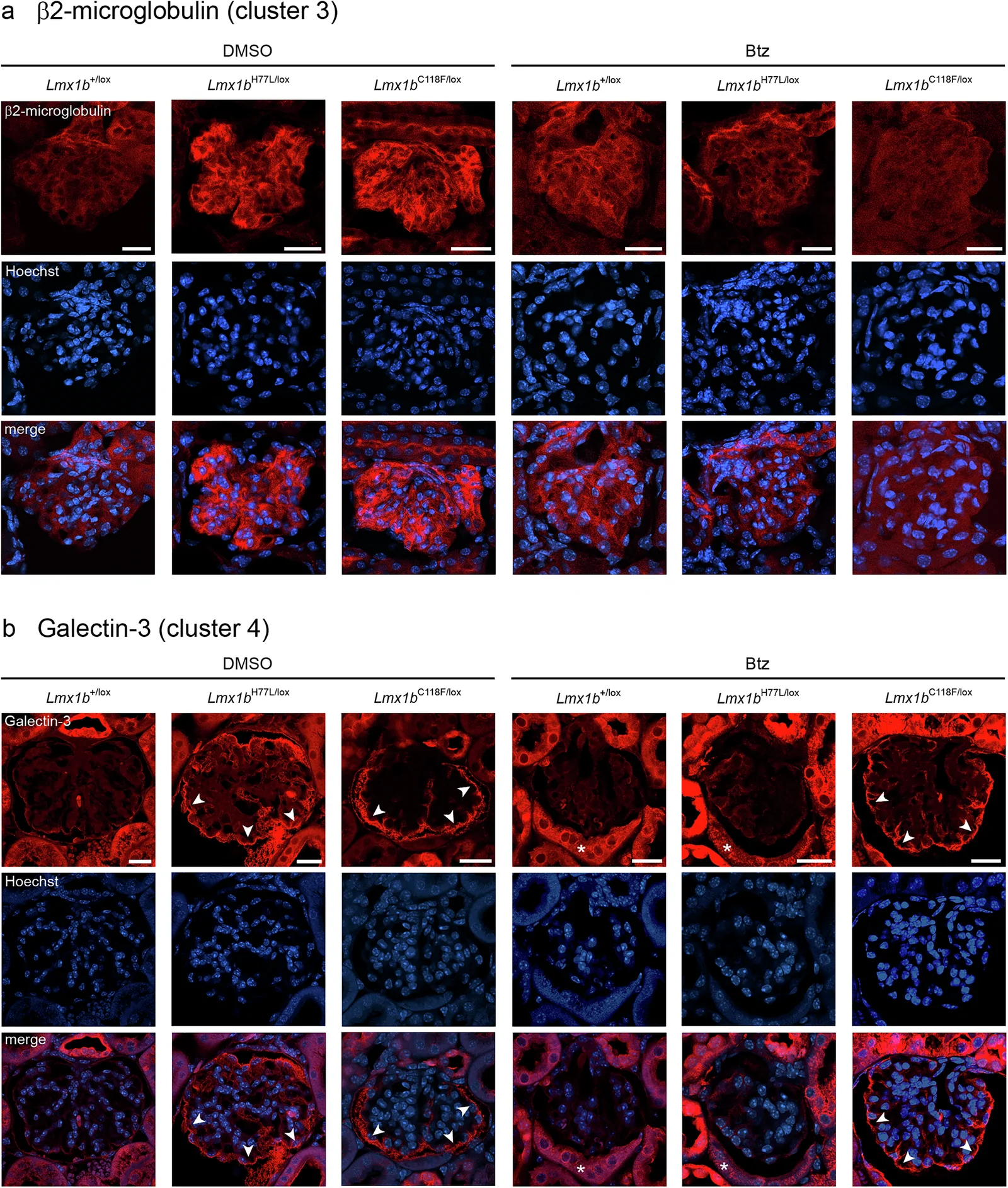
Protein expression pattern after treatment of *Lmx1b*^H77L/lox^ and *Lmx1b*^C118F/lox^ knock-in mice with DMSO and bortezomib. *Lmx1b*^+/lox^, *Lmx1b*^H77L/lox^ and *Lmx1b*^C118F/lox^ mice with a podocyte-specific, doxycycline-inducible expression cassette for Cre recombinase (*Lmx1b*^+/lox^ / *NPHS2*::rtTA / tetO::Cre mice; *Lmx1b*^H77L/lox^ / *NPHS2*::rtTA / tetO::Cre mice; *Lmx1b*^C118F/lox^ / *NPHS2*::rtTA / tetO::Cre mice) were analyzed. Mice were between 7–10 weeks of age. Induction of Cre recombinase was started on day 0 by the administration of doxycycline in the drinking water together with the intraperitoneal injection of DMSO (solvent for bortezomib) or bortezomib every other day. On day 7, mice were perfusion-fixed, and kidney sections were stained with the respective antibodies. Nuclei were counter-stained with Hoechst 33258 (Hoechst). Three mice were analyzed for each genotype and treatment, a representative result in each case is shown. **a** No staining for β2-microglobulin is detectable in the podocytes of *Lmx1b*^+/lox^ mice, but the protein is strongly upregulated in both DMSO-treated, but not in the bortezomib-treated, knock-in lines. **b** Galectin-3 is absent in the podocytes of *Lmx1b*^+/lox^ mice, but its expression strongly increases in both DMSO-treated knock-in lines. This increase is prevented after bortezomib treatment in the *Lmx1b*^H77L/lox^, but not the *Lmx1b*^C118F/lox^, line. Arrow heads point to positive podocytes. Asterisks mark the proximal tubule of the urinary pole. *Lmx1b*^+/lox^, *Lmx1b*^+/lox^ / *NPHS2*::rtTA / tetO::Cre / mTmG mice; *Lmx1b*^H77L/lox^, *Lmx1b*^H77L/lox^ / *NPHS2*::rtTA / tetO::Cre / mTmG mice; *Lmx1b*^C118F/lox^, *Lmx1b*^C118F/lox^ / *NPHS2*::rtTA / tetO::Cre / mTmG mice. Scale bars, 20 μm.

Additional genes were examined by immunofluorescence staining kidney sections of *Lmx1b*^+/lox^ / *NPHS2*::rtTA / tetO::Cre, *Lmx1b*^H77L/lox^ / *NPHS2*::rtTA / tetO::Cre and *Lmx1b*^C118F/lox^ / *NPHS2*::rtTA / tetO::Cre mice treated with DMSO and bortezomib. Cluster 1 contains genes whose expression is down-regulated in the knock-in mice independently of the administration of DMSO and bortezomib. Both the cyclin-dependent kinase inhibitor 1c (Cdkn1c, also known as p57)^30,31^ and claudin-5^32,33^ have been shown to be present in the podocytes of normal kidneys. They are representative of cluster 1 and, in accordance with the previous reports, were present in the non-damaged podocytes of *Lmx1b*^+/lox^ / *NPHS2*::rtTA / tetO::Cre both after treatment with DMSO and bortezomib. Consistent with the RNA sequencing data, we noticed a markedly lower number of p57-positive nuclei (Fig. 9a) and of claudin-5 (Supplementary Fig. 8a) in both knock-in lines independently of bortezomib treatment. Transgelin (also known as SM22α) is virtually absent in healthy podocytes^34,35^, it belongs to cluster 2 where an upregulation is predicted in both knock-in mouse lines independently of bortezomib treatment. Indeed, this is what we observed on kidney sections (Fig. 9b). The genes in cluster 3 are characterized by higher mRNA levels in both DMSO-treated knock-in mouse lines, but not after bortezomib treatment. Immunofluorescence staining for β2-microglobulin confirmed this pattern (Fig. 10a). Finally, cluster 4 contains genes whose mRNA levels rise in both knock-in lines, but this increase is prevented by bortezomib treatment in the *Lmx1b*^H77L/lox^ / *NPHS2*::rtTA / tetO::Cre mice. Galectin-3 is not present in healthy podocytes but appears after various forms of podocyte injury^36,37^. Similarly, the Cd63 antigen is upregulated in focal segmental glomerulosclerosis^38^. For both galectin-3 (Fig. 10b) and the Cd63 antigen (Supplementary Fig. 8b), we detected staining in the DMSO-treated *Lmx1b*^H77L/lox^ and *Lmx1b*^C118F/lox^ mouse lines, which was absent in the bortezomib-treated *Lmx1b*^H77L/lox^ but still present in the *Lmx1b*^C118F/lox^ mouse line.

## Discussion

In patients suffering from nail-patella syndrome a variety of mutations have been identified but the knowledge regarding the underlying pathogenetic mechanisms is rather scarce. Therefore, we designed in vivo and in vitro experiments to investigate how mutations in the LIM domains of LMX1B lead to podocyte damage. Our *Lmx1b*^H77L^ and *Lmx1b*^C118F^ knock-in mice mimic mutations observed in patients where zinc-chelating amino acids have been mutated in one of the two LIM domains of Lmx1b. Contrary to our expectation, the hypothesis that the mutated proteins act in a dominant-negative fashion because they can still bind to their target genes via their homeodomain, but cannot interact with other proteins because one of their LIM domains is defective, turned out to be wrong. Instead, we found that both mutants were less stable than the wild-type Lmx1b protein, which once again argues for a loss of function as the pathogenetic mechanism. Probably the loss of one zinc-chelating amino acid leads to a misfolding of the LIM domain, which is recognized by the cellular quality control machinery and subsequently leads to the ubiquitin-mediated degradation of misfolded Lmx1b in the proteasome.

Whereas nail-patella syndrome is inherited in an autosomal-dominant fashion^1^, all evidence obtained so far supports the notion that in mice both *Lmx1b* alleles have to be inactivated for a phenotype to develop. With respect to the kidneys, neither the inactivation of *Lmx1b* in conventional knock-out mice^5,6^ nor the constitutive podocyte-specific inactivation of *Lmx1b*^7^ or the inducible podocyte-specific inactivation of *Lmx1b* in adult mice^8^ yields a phenotype in the heterozygous state. Consequently, at least with respect to mutations in the LIM domains, we still lack an explanation for the discrepancy concerning the genetics in patients and in mice. The renal symptoms, which affect only 40% of patients with nail-patella syndrome, vary greatly in severity and take years to develop^1^, could be explained by environmental effects damaging the podocytes and/or by somatic mutations in the *LMX1B* gene accumulating in podocytes over the years (two-hit mechanism). It is unlikely that such a prolonged pathogenetic mechanism can be observed in mice due to their short life span. Another explanation could lie in the fact that we have maintained our mice on a C57BL/6 genetic background since mice on this background are known to be more resistant to renal damage than other strains. However, the limb defects such as the ventralization of the paws or the absence of patellae cannot be caused by somatic mutations because the defect has to be present already *in utero* - either limbs develop normally, or they do not, and once they are formed properly, the anatomy will not change. On the other hand, the pathogenetic mechanism resulting from mutations in the DNA-binding homeodomain is consistent between the genetics in patients and with previous mouse data^21^, as well as our current in vitro results. Apparently, the LMX1B^V265D^ mutant protein, in which a highly conserved valine residue is replaced by an aspartate residue, is even more stable than the wild-type protein. According to our modeling, the LMX1B^V265D^ mutant is predicted to bind less tightly to LMX1B target genes, which offers an explanation for its dominant effect. Therefore, one important result of our study is the conclusion that the way in which nail-patella syndrome develops on a biochemical basis depends on the nature of the mutation in the *LMX1B* gene, with different pathways ultimately leading to the disease.

This conclusion has important implications when it comes to designing therapeutic strategies for patients with nail-patella syndrome whose prognosis is determined by their renal symptoms^1^. Currently, no therapy can be offered to them, but our results with bortezomib provide proof of principle that proteasomal inhibition may be a strategy to prevent the progression of podocyte damage. Bortezomib is a promising substance because a majority of mutations in the *LMX1B* gene cluster in the region encoding the LIM domains^4^ and because bortezomib and other proteasomal inhibitors are already being used for treating patients with multiple myeloma^23^. In the case of nail-patella syndrome it may take decades before kidney symptoms or even end-stage renal disease develop^1^, which means that treatment with bortezomib would not have to start at birth. Nevertheless, the continuous administration of bortezomib over many years probably will have considerable side effects because podocytes in particular - as highly differentiated, non-dividing cells - depend on an intact protein degradation machinery^39^. Therefore, it would be very beneficial to develop a screening procedure to differentiate between patients who will have only a minimal or no decline in kidney function and those who will require dialysis or a transplant. Furthermore, no data are available to determine whether the deterioration of kidney function in patients with nail-patella syndrome proceeds steadily or intermittently, in the latter case bortezomib would have to be given only in periods of active disease. An alternative option to cut down on side effects would be to deliver bortezomib specifically to podocytes, e.g., by encapsulating the drug into nanoparticles targeting podocytes. Regrettably, such nanoparticles are not available yet. Finally, it has to be remembered that the ubiquitinylation of proteins proceeds in a stepwise fashion via the action of, at first, ubiquitin-activating enzymes, then ubiquitin-conjugating enzymes and finally ubiquitin ligases. Ubiquitin ligases are a large family of enzymes which target distinct proteins and are present in distinct cell types, in contrast to the proteasome with its ubiquitous expression pattern. Therefore, yet another option lies in the inhibition of the - still to be identified - ubiquitin ligase(s) by which LMX1B is ubiquitinylated. In the case of mutations in the homeodomain, such as the V265D missense mutation, a different therapeutic approach is called for because the mutant protein is more stable and probably binds less tightly to DNA than the wild-type protein. Here, it may be necessary to destabilize the mutant proteins.

A surprising outcome of our RNAseq data concerns the fact that approximately four times more genes were up- than down-regulated in both *Lmx1b*^H77L/lox^ and *Lmx1b*^C118F/lox^ knock-in mice. Generally, LMX1B is considered to act as a transcriptional activator^5,6,8^ which accordingly should have led to a down-regulation of genes in the compound heterozygous mice. It is remarkable that *Irf7*, which is upregulated in both *Lmx1b* compound heterozygous mouse lines, is also upregulated in the conventional *Lmx1b* knock-out mice^40^, and that the GO analysis identified interferon-regulated genes (‘response to interferon-beta’) as a family of putative Lmx1b target genes, similar to our previous findings^40^. Further experiments will have to be carried out to determine whether LMX1B acts as a transcriptional activator or repressor or both, possibly depending on the context of the promoter it binds to. It also remains an open question whether the two LIM domains in LMX1B are of equivalent importance in regulating any target gene or whether the LIM domains exert gene-specific functions. The preliminary analysis of the podocyte-specific RNAseq data coming from our *Lmx1b*^H77L/lox^ and *Lmx1b*^C118F/lox^ knock-in mice offers some support for the second alternative. By stabilizing the mutated Lmx1b proteins with bortezomib, they can still bind to their target genes, but now only one LIM domain will mediate the interaction with other proteins. It is surprising that in our experimental setting, only one functional LIM domain is sufficient for Lmx1b to serve its function in podocytes because the stabilization of both Lmx1b^H77L^ and Lmx1b^C118F^ ameliorated proteinuria. Nevertheless, there may be specific effects because we identified several genes whose levels were dysregulated in both compound heterozygous mouse lines but were normalized at least to some extent by bortezomib treatment only in the *Lmx1b*^H77L/lox^ mice. According to the following scenario, we interpret this finding such that the regulation of the aforementioned genes depends on an intact LIM-B domain. Without bortezomib, both mutant proteins are destabilized, and their levels are too low to serve their physiological role (regardless of whether they are still functional or not). When the Lmx1b^H77L^ mutant becomes stabilized upon bortezomib treatment, the interaction with other proteins will be mediated exclusively by the LIM-B domain because the LIM-A domain is misfolded. On the other hand, when the Lmx1b^C118F^ protein becomes stabilized, its LIM-B domain is misfolded and cannot mediate the interaction with other proteins. A more in-depth analysis will be required to find out whether any genes exist whose regulation in podocytes depends exclusively on an intact LIM-A domain.

## Methods

### Mouse strains

The targeting constructs harboring the knock-in mutations for the Lmx1b^H77L^ and Lmx1b^C118F^ proteins were custom-made by Gene Bridges (Heidelberg, Germany) using the Red/ET recombination system. A neomycin resistance cassette with flanking FRT sites was introduced in the intron between exons 2 and 3 again using Red/ET recombination. The final targeting constructs were used to transfect embryonic stem cells. Once germline transmission was established, the neomycin resistance cassette was removed by mating the two knock-in mouse lines with the FLP deleter mouse line TGM/hACTB-FLPe^41^. All heterozygous offspring were backcrossed to C57Bl/6 J mice. For genotyping, PCR of genomic DNA isolated from tail biopsies was performed with the primer pairs 5’-AAA GGA GCG TGC TGT CTA GG-3’ and 5’-GCT GGA AGG AGA TGA TTC G-3’ for the *Lmx1b*^H77L^ line and 5’-GTT TGG CTA TGG GGT TGA GG-3’ and 5’-CAT AGT CAC CCT TGC ACA GC-3’ for the *Lmx1b*^C118F^ line. For identification of the mutations in the Lmx1b mRNA, total RNA was isolated from E18.5 wild-type, *Lmx1b*^H77/H77L^ and *Lmx1b*^C118F/C118F^ embryos and a RT-PCR carried out with the oligonucleotides 5’-ATC AAG ATG GAG GAG CAC GC-3’ and 5’-GAG TCG TTC CCT GGC ATT TG-3’.

Podocyte-specific synthesis of the reverse tetracycline-controlled transcriptional transactivator (rtTA) in a transgenic mouse line was achieved through a 2.5 kbp fragment of the human *NPHS2* gene (*NPHS2*::rtTA mice)^42^. Doxycycline-dependent synthesis of Cre recombinase was obtained by mating the *NPHS2*::rtTA mice with a transgenic mouse line containing an expression cassette with the Cre cDNA under control of tet operator elements (tetO::Cre mice, also known as LC-1 mice)^43^. Floxed *Lmx1b* knock-out mice contain loxP sites upstream and downstream of the exons encoding the homeodomain of Lmx1b (exons 4 and 6, respectively) and were kindly provided by Randy Johnson^7^. Transgenic mice with ubiquitous synthesis of the red fluorescent mTomato protein and Cre-mediated synthesis of the green fluorescent EGFP protein (mT/mG mice)^24^ allowed the specific sorting of podocytes.

Breeding and genotyping were carried out following standard procedures. Mice were housed in a specific pathogen-free facility. The animal experiments were conducted according to the guidelines of the German law for the welfare of animals and were approved by local authorities (Regierung von Unterfranken, Az. 55.2.2 2532.2-1170).

### Whole-mount skeletal staining and imaging via μCT analysis

Embryos were collected at 18.5 days of gestation in 1x PBS pH 7.4 at room temperature. Extraembryonic membranes lining the embryo were removed, and photographs of the foot pads were taken. For skeletal staining, the skin was removed, and embryos were fixed in 95% ethanol for 2 days at 4 °C under constant agitation. Then the ethanol was replaced by acetone, and the embryos were incubated overnight at room temperature under constant agitation. After several washing steps with 95% ethanol, alcian blue staining of cartilage and subsequent alizarin red staining of bones were performed following standard protocols.

For μCT analysis, embryos were collected at 18.5 days of gestation and fixed in 95% ethanol as described above. All scans were performed on the micro-CT system phoenix v|tome|x s 240/180 research edition from Baker Hughes Digital Solutions GmbH (software phoenix datos|x 2 acquisition 2.5.1). Scanning parameters were as follows: 23 kV voltage, 600 μA current, 500 ms time, 1000 images, voxel size 30–35 μm. Reconstructed volumes were processed using the manufacturer’s software phoenix datos|x 2 reconstruction 2.5.1. Three-dimensional images were created using the software Volume Graphics VG Studio Max.

### Perfusion-fixation and histological examination of the kidneys

Mice were perfusion-fixed through the distal abdominal aorta with 4% paraformaldehyde, 1x PBS for three minutes. Kidneys were taken out, cut in half and further incubated at 4 °C at least overnight with either 2% glutaraldehyde, 50 mM cacodylate for electron microscopy, with 4% paraformaldehyde, 1x PBS for paraffin embedding or with 18% sucrose, 1x PBS for immunofluorescence microscopy.

For transmission electron microscopy, kidneys were incubated in 1% OsO_4_, 50 mM cacodylate for 2-3 h before embedding in epon. Ultrathin epon sections (50 nm) were prepared with an ultramicrotome (Leica EM UC6), stained with uranyl acetate plus lead citrate and visualized in a transmission electron microscope (Zeiss EM 902).

For histological stainings, the kidneys were embedded in paraffin. Six μm thick paraffin sections were stained with hematoxylin and eosin.

For immunofluorescence staining, kidneys were embedded in Tissue-Tek^®^ and paraffin. Twelve μm thick cryosections were cut in a cryotome (Cryostar NX70). Sections were dehydrated with cold ethanol for 10 minutes, washed 3 times with 1x PBS pH 7.5, blocked for 30 minutes with 1x PBS, 1% bovine serum albumin (BSA), 10% horse serum, and incubated overnight at 4 °C with the primary antibody. The next morning, sections were washed three times with 1x PBS before the secondary antibody was added and the incubation continued for 45 min at room temperature. Sections were then stained for 1 min with the Hoechst dye 33258 (0.2 μg/ml), washed three times with 1x PBS and mounted. Six μm thick paraffin-embedded sections were cut in a microtome (Leica RM2255), deparaffinized and subjected to antigen retrieval for 2 h at 95 °C in 10 mM sodium citrate, pH 6.0. The subsequent treatment was the same as for the cryosections. Pictures were taken with a confocal laser scanning microscope (Zeiss LSM 710 and Zeiss LSM 980).

### Expression plasmids, and generation of transfected and transduced cells

The full-length human LMX1B cDNA with a DNA fragment encoding the myc-epitope at its COOH-terminus was cloned into the mammalian expression plasmid pUHD 10-3^44^, and into pInducer21^45^ to generate recombinant lentiviruses. Plasmids for the expression of the LMX1B^H77L^, LMX1B^C118F^ and LMX1B^V265D^ mutant proteins were generated by site-directed mutagenesis with oligonucleotides containing the desired mutations. The recombinant pUHD 10-3 expression plasmids were stably transfected into HeLa cells, synthesizing the tetracycline-dependent transactivator (HtTA-1 cells)^44^ in which expression was shut off in the presence of 100 ng/ml of doxycycline. Recombinant lentiviruses were used to transduce an immortalized human podocyte cell line^46^ in which expression was turned on in the presence of 1 μg/ml of doxycycline.

### qPCR analysis and determination of protein half-lives

qPCR analyses were performed using the SensiFAST™ SYBR® No-ROX Kit® according to the instructions of the manufacturer (Bioline / Meridian Bioscience), lamin mRNA levels were used for normalization.

Cell lines were induced for the indicated times before cycloheximide was added at a final concentration of 100 μg/ml. Where applicable, bortezomib, MG132 or bafilomycin A_1_ were added simultaneously at a final concentration of 10 μM. Lysates were prepared by the addition of 600 μl of lysis buffer containing 1x PBS pH 7.4, 6 M urea, 1% Triton X-100 to the cell pellet after 0, 0.5, 1, 1.5, 2, 4, 8, 12, 14 and 24 h. Then 40 μg of total protein were subjected to SDS-polyacrylamide gel electrophoresis and Western blot analysis with the anti-LMX1B antibody BMO8^7^.

### Immunoprecipitation of ubiquitinylated proteins

The respective cell lines were transiently transfected with an expression plasmid encoding a fusion protein of 7 concatemerized ubiquitin molecules tagged with a HA-epitope at the NH_2_-terminus. Forty-eight hours later cells were lysed with 1x PBS pH 7.4, 1% Triton X-100 in the presence of the protease inhibitors 4-(2-Aminoethyl)benzolsulfonylfluorid (AEBSF), aprotinin and leupeptin, N-ethylmaleimide was added to inhibit deubiquitylases^47^. Protein A/G Plus Agarose beads (Santa Cruz Biotechnology) were washed with lysis buffer before being incubated overnight at 4 °C with 1.2 ml of supernatant from the murine hybridoma producing the monoclonal anti-HA antibody 12CA5. The next day the beads were washed with lysis buffer three times and incubated for three hours with cell lysates containing 400 μg of total protein. Beads were washed again with lysis buffer, resuspended in 1x reducing sample buffer and boiled for 5 min. Supernatants were used for protein gel electrophoresis and Western blotting, followed by specific detection of the desired proteins with the rat monoclonal anti-HA antibody 3F10 (diluted 1:1000; Roche, cat. no. 11 867 423 001) and the anti-LMX1B antibody BMO8^7^.

### Calculation of the electrostatic potential of the wild-type and V265D mutant homeodomain

Electrostatic potential calculations were performed using the Adaptive Poisson-Boltzmann Solver (APBS) server (version 3.4.1). PQR files, generated from PDB2PQR at pH 7.0 with protonation states assigned by PROPKA^48,49^, were used as input. The protein interior dielectric constant was set to 2.0, while the solvent dielectric constant was set to 78.5, representing water at 298.15 K, with a solvent probe radius of 1.4 Å^50^. ChimeraX^51^ was used for visualization and analysis of the electrostatic potential maps.

### Estimation of proteinuria on Coomassie-stained protein gels

For semi-quantitative visualization of proteinuria, 1 μl of urine was diluted 5fold with distilled water, then 2 μl of the diluted urine were combined with 6 μl distilled water and 2 μl of 5x Laemmli sample buffer. After boiling for 5 min, samples were run on 8% polyacrylamide gels alongside increasing amounts of bovine serum albumin. Proteins were visualized by staining with Coomassie Brilliant Blue R250 for 30 min.

### Measurement of protein and creatinine concentrations via Bradford assay and Jaffe´s reaction, respectively

One μl of each urine sample was diluted 50fold with distilled water before being added either to 200 μl of 1.2x Roti^®^Quant for protein measurements or to 200 μl of a 35 mM picric acid solution for creatinine measurements in 96-well plates. After incubating the samples for 5 min, measurements were carried out in a Tecan Plate Reader at wavelengths of 450 and 590 nm using the Tecan Sunrise Software Magellan 7.2 to determine the protein concentration. For creatinine measurements, samples were incubated for 30 min and absorption was measured at 540 nm.

### Podocyte-specific inactivation of the Lmx1b gene and treatment with bortezomib

Podocyte-specific inactivation of the *Lmx1b* gene in mice was achieved through the administration of 2 mg/ml of doxycycline to the drinking water for a period of seven days. A stock solution of bortezomib was prepared at a concentration of 1 μg/μl in DMSO, the required amounts for injections were combined with a volume of 1x PBS corresponding to 1% of the body weight. Beginning with the administration of doxycycline, 0.3 μg of bortezomib per g body weight were injected intraperitoneally every other day, the solvent DMSO served as a negative control. Spot urine was collected from the mice on day 7 before they were subjected to perfusion-fixation under general anesthesia with xylazine/ketamine.

### Isolation of podocytes and RNA extraction

Genetically modified mice with podocyte-specific expression of the reverse tetracycline-controlled transcriptional transactivator (*NPHS2*::rtTA mice), the doxycycline-dependent synthesis of Cre recombinase (LC-1 mice), and Cre-mediated synthesis of the green fluorescent EGFP protein (mT/mG mice) together with the respective mutations in the *Lmx1b* gene were used for the specific isolation of podocytes. Both kidneys together with neighboring stretches of the abdominal aorta were removed and perfused with suspensions of M-450 tosylactivated magnetic dynabeads (ThermoFisher Scientific, cat. no. 14013) in 1x HBSS pH 7.5. Podocytes were isolated according to a previously described protocol with slight modifications^52,53^. After the final digest, cell suspensions were passed through a 40 μm cell strainer and subjected to FACS. Green fluorescent podocytes were collected in RNA*later*^®^ and then processed further in the Kompetenzzentrum Fluoreszente Bioanalytik (KFB) using the RNeasy Plus Micro kit (Qiagen).

### RNA sequencing and gene expression profiling

To determine the number of replicates required for differential RNA sequencing analysis, a statistical power analysis was performed using the R package ssizeRNA^54^. The expected dispersion was calculated using a similar RNAseq dataset of isolated podocytes (GSE24922)^55^. Statistical power analysis revealed an 80–86% power at a false discovery rate of 5% to detect genes with a fold-change of two using five to six replicates. Single-end polyA-enriched RNA-seq data of *Lmx1b*^H77L/lox^ and *Lmx1b*^C118F/lox^ podocytes treated either with DMSO or bortezomib were annotated using an in-house Nextflow RNA-seq pipeline^56,57^ (available at GitHub: https://github.com/uschwartz/RNAseq_NAC; v2.1). Initially, quality control of the raw sequence reads was conducted using FastQC (v0.11.8)^58^. Adapter sequences were trimmed from the 3’ ends using Trim Galore (v0.6.7)^59^. Reads were mapped to the mouse reference genome (GRCm39) and the corresponding gene annotation (Ensembl version 110) using Spliced Transcripts Alignment to a Reference (STAR v2.7.8a)^60^. The following options were used to optimize the alignment process: --outFilterType BySJout, --outFilterMultimapNmax 20, --alignSJoverhangMin 8, --alignSJDBoverhangMin 1, --outFilterMismatchNmax 999, --alignIntronMin 10, --alignIntronMax 1,000,000, --outFilterMismatchNoverReadLmax 0.04, --runThreadN 12, --outSAMtype BAM SortedByCoordinate, --outSAMmultNmax 1, and --outMultimapperOrder Random. Postmapping quality control was performed using the RNAseq analysis mode of Qualimap (v2.2.1)^61^. The level of PCR duplication was assessed using Picard MarkDuplicates (v2.21.8)^62^ and dupRadar (v1.15.0)^63^. Gene expression quantification for protein-coding genes was carried out using featureCounts (v1.6.3)^64^.

### Differential gene expression analysis

Differential gene expression analysis between either *Lmx1b*^H77L/lox^ or *Lmx1b*^C118F/lox^ and *Lmx1b*^+/lox^ samples was performed using the Bioconductor DESeq2 package^65^. The ashr shrinkage method was applied to properly scale log_2_(fold-changes)^66^. Differentially expressed genes were identified using a false discovery rate of 0.001 and a | log_2_(fold-change) | > 2 as cutoffs, with at least one condition requiring all samples to have an abundance of greater than 1 transcript per million.

### Clustering analysis

For clustering analysis, all genes which were differentially regulated under DMSO treatment in either *Lmx1b*^H77L/lox^ or *Lmx1b*^C118F/lox^ mice compared to the control group of *Lmx1b*^+/lox^ mice under DMSO treatment, respectively, were considered. The median rlog value of each group, including bortezomib-treated samples, was extracted for each gene. Next, the median rlog values were scaled to *z* scores across all conditions and split into four distinct clusters using k-means clustering within the pheatmap function in R^67^. Examples from clusters with patterns showing a significant response in regulation after bortezomib treatment, when contrasted to values under DMSO treatment, were visualized as box plots using ggpubr^68^. Here, transcript per million values of *Lmx1b*^H77L/lox^ and *Lmx1b*^C118F/lox^ samples were used, and the fold change to the median transcript per million value of the corresponding control group was calculated. To account for problems with genes which were not expressed or expressed at very low levels a pseudocount of 1 was added.

### Gene enrichment analysis

The differentially expressed genes were split into genes whose expression levels had changed significantly in the *Lmx1b*^H77L/lox^ and *Lmx1b*^C118F/lox^ mice or within their respective clusters before being used for gene ontology (GO) analysis against all expressed genes in Metascape^69^. Here, a *p-*value of 0.01 and a minimum overlap of 5 genes were used as cutoffs.

### Statistical analysis

For statistical evaluation, groups were compared to each other using Student’s *t* test implemented into Excel. When necessary, a Bonferroni correction was applied.

### Reporting summary

Further information on research design is available in the Nature Portfolio Reporting Summary linked to this article.

## Supplementary information

Reporting Summary

Supplementary Information

Transparent Peer Review file

## Source data

Source data

## Data Availability

Raw and processed sequencing data have been deposited to the NCBI Gene Expression Omnibus (GEO) database and assigned the identifier GSE288803 (https://www.ncbi.nlm.nih.gov/geo/query/acc.cgi?acc=GSE288803). Source data are provided in this paper.

## Code availability

The RNAseq pipeline (v2.1) used in this paper is available at GitHub under https://github.com/uschwartz/RNAseq_NAC. The code used to analyze the data is available at GitHub under https://github.com/uschwartz/lmx1b_dysfunctional_lim_domain.
