# Supplementary material for: Proteasome inhibition alleviates proteinuria in Lmx1b knock-in mice with dysfunctional LIM domains
Source: Nat Commun. 2026 Sep 11;17:9731. doi: 10.1038/s41467-026-77485-1 (PMC13562594; doi:10.1038/s41467-026-77485-1)
Supplement: Supplementary file 1 [file 41467_2026_77485_MOESM1_ESM.pdf]

nature portfolio

Corresponding author(s): Ralph Witzgall

Last updated by author(s): Aug 18, 2026

Reporting Summary

Nature Portfolio wishes to improve the reproducibility of the work that we publish. This form provides structure for consistency and transparency in reporting. For further information on Nature Portfolio policies, see our Editorial Policies and the Editorial Policy Checklist.

Statistics

For all statistical analyses, confirm that the following items are present in the figure legend, table legend, main text, or Methods section.

n/a	Confirmed	
☐	☒	The exact sample size (*n*) for each experimental group/condition, given as a discrete number and unit of measurement
☐	☒	A statement on whether measurements were taken from distinct samples or whether the same sample was measured repeatedly
☐	☒	The statistical test(s) used AND whether they are one- or two-sided<br>*Only common tests should be described solely by name; describe more complex techniques in the Methods section.*
☒	☐	A description of all covariates tested
☐	☒	A description of any assumptions or corrections, such as tests of normality and adjustment for multiple comparisons
☐	☒	A full description of the statistical parameters including central tendency (e.g. means) or other basic estimates (e.g. regression coefficient) AND variation (e.g. standard deviation) or associated estimates of uncertainty (e.g. confidence intervals)
☐	☒	For null hypothesis testing, the test statistic (e.g. *F*, *t*, *r*) with confidence intervals, effect sizes, degrees of freedom and *P* value noted<br>*Give P values as exact values whenever suitable.*
☒	☐	For Bayesian analysis, information on the choice of priors and Markov chain Monte Carlo settings
☒	☐	For hierarchical and complex designs, identification of the appropriate level for tests and full reporting of outcomes
☒	☐	Estimates of effect sizes (e.g. Cohen's *d*, Pearson's *r*), indicating how they were calculated

Our web collection on statistics for biologists contains articles on many of the points above.

Software and code

Policy information about availability of computer code

Data collection	To determine the number of replicates required for differential RNA sequencing analysis, a statistical power analysis was performed using the R package ssizeRNA. The expected dispersion was calculated using a similar RNAseq dataset of isolated podocytes (GSE24922). Statistical power analysis revealed a 80-86% power at a false discovery rate of 5% to detect genes with a fold-change of two using five to six replicates.
Data analysis	Single-end polyA-enriched RNA-seq data were annotated using an in-house nextflow RNA-seq pipeline (available at GitHub: https://github.com/uschwartz/RNAseq_NAC; v2.1). Initially, quality control of the raw sequence reads was conducted using FastQC (v0.11.8). Adapter sequences were trimmed from the 3' ends using Trim Galore (v0.6.7). Reads were mapped to the mouse reference genome (GRCm39) and the corresponding gene annotation (Ensembl version 110) using Spliced Transcripts Alignment to a Reference (STAR v2.7.8a).<br><br>Postmapping quality control was performed using the RNAseq analysis mode of Qualimap (v2.2.1). The level of PCR duplication was assessed using Picard MarkDuplicates (v2.21.8) and dupRadar (v1.15.0). Gene expression quantification for protein-coding genes was carried out using featureCounts (v1.6.3).<br><br>Differential gene expression analysis was performed using the Bioconductor DESeq2 package. The ashr shrinkage method was applied to properly scale log2(fold-changes). Differentially expressed genes were identified using a false discovery rate of 0.001 and a | log2(fold-change) | > 2 as cutoffs, with at least one condition requiring all samples to have an abundance of greater than 1 transcript per million.<br><br>For clustering analysis, all genes which were differentially regulated were considered. The median rlog value of each group including bortezomib-treated samples was extracted for each gene. Next the median rlog values were scaled to z scores across all conditions and split into four distinct clusters using k-means clustering within the pheatmap function in R 67. Examples from clusters with patterns showing a significant response were visualized as box plots using ggpubr. Here transcript per million values were used and the fold change to the median

transcript per million value of the corresponding control group was calculated. To account for problems with genes which were not expressed or expressed at very low levels a pseudocount of 1 was added.

For manuscripts utilizing custom algorithms or software that are central to the research but not yet described in published literature, software must be made available to editors and reviewers. We strongly encourage code deposition in a community repository (e.g. GitHub). See the Nature Portfolio guidelines for submitting code & software for further information.

Data

Policy information about availability of data

All manuscripts must include a data availability statement. This statement should provide the following information, where applicable:

- Accession codes, unique identifiers, or web links for publicly available datasets
- A description of any restrictions on data availability
- For clinical datasets or third party data, please ensure that the statement adheres to our policy

The RNAseq pipeline (v2.1) used in this paper is available at Github (https://github.com/uschwartz/RNAseq_NAC). Raw and processed sequencing data have been deposited to the NCBI Gene Expression Omnibus (GEO) database and assigned the identifier GSE288803. There are no restrictions on data availability.

Research involving human participants, their data, or biological material

Policy information about studies with human participants or human data. See also policy information about sex, gender (identity/presentation), and sexual orientation and race, ethnicity and racism.

Reporting on sex and gender	*Use the terms sex (biological attribute) and gender (shaped by social and cultural circumstances) carefully in order to avoid confusing both terms. Indicate if findings apply to only one sex or gender; describe whether sex and gender were considered in study design; whether sex and/or gender was determined based on self-reporting or assigned and methods used. Provide in the source data disaggregated sex and gender data, where this information has been collected, and if consent has been obtained for sharing of individual-level data; provide overall numbers in this Reporting Summary. Please state if this information has not been collected. Report sex- and gender-based analyses where performed, justify reasons for lack of sex- and gender-based analysis.*
Reporting on race, ethnicity, or other socially relevant groupings	*Please specify the socially constructed or socially relevant categorization variable(s) used in your manuscript and explain why they were used. Please note that such variables should not be used as proxies for other socially constructed/relevant variables (for example, race or ethnicity should not be used as a proxy for socioeconomic status). Provide clear definitions of the relevant terms used, how they were provided (by the participants/respondents, the researchers, or third parties), and the method(s) used to classify people into the different categories (e.g. self-report, census or administrative data, social media data, etc.) Please provide details about how you controlled for confounding variables in your analyses.*
Population characteristics	*Describe the covariate-relevant population characteristics of the human research participants (e.g. age, genotypic information, past and current diagnosis and treatment categories). If you filled out the behavioural & social sciences study design questions and have nothing to add here, write "See above."*
Recruitment	*Describe how participants were recruited. Outline any potential self-selection bias or other biases that may be present and how these are likely to impact results.*
Ethics oversight	*Identify the organization(s) that approved the study protocol.*

Note that full information on the approval of the study protocol must also be provided in the manuscript.

Field-specific reporting

Please select the one below that is the best fit for your research. If you are not sure, read the appropriate sections before making your selection.

☒ Life sciences ☐ Behavioural & social sciences ☐ Ecological, evolutionary & environmental sciences

For a reference copy of the document with all sections, see nature.com/documents/nr-reporting-summary-flat.pdf

Life sciences study design

All studies must disclose on these points even when the disclosure is negative.

Sample size	Our study reports completely new biochemical, clinical chemical, light and electron microscopic data for Lmx1b knock-in mice. Since we did not know what to expect and there was no precedent in the literature, It was impossible to estimate the size effects and accordingly calculate the necessary sample sizes. From our experience with other genetically altered mouse models and biochemical experiments we considered a size of at least 4 or 5 independent biological samples or experiments as sufficient.<br><br>To determine the number of replicates required for differential RNA sequencing analysis, a statistical power analysis was performed using the R package ssizeRNA. The expected dispersion was calculated using a similar RNAseq dataset of isolated podocytes (GSE24922). Statistical power analysis revealed a 80-86% power at a false discovery rate of 5% to detect genes with a fold-change of two using five to six replicates.

Data exclusions	No data were excluded from the analysis.
Replication	Whenever experiments were carried out multiple times these experiments were not performed on the same day but rather on different days and in different weeks. The cells required for different experiments were plated out independently, mice were taken from different litters. Naturally, the results reported in our manuscript varied to some extent as shown by the individual data points but they were reproducible.
Randomization	Mice of the required genotypes were randomly chosen from different litters.
Blinding	For the quantification of podocyte cell-cell contacts, ultrathin kidney sections were subjected to transmission electron microscopy. Subsequently the number of cell-cell contacts were counted in a blinded fashion.

Reporting for specific materials, systems and methods

We require information from authors about some types of materials, experimental systems and methods used in many studies. Here, indicate whether each material, system or method listed is relevant to your study. If you are not sure if a list item applies to your research, read the appropriate section before selecting a response.

Materials & experimental systems

n/a	Involved in the study	
☐	☒	Antibodies
☐	☒	Eukaryotic cell lines
☒	☐	Palaeontology and archaeology
☐	☒	Animals and other organisms
☒	☐	Clinical data
☒	☐	Dual use research of concern
☒	☐	Plants

Methods

n/a	Involved in the study	
☒	☐	ChIP-seq
☒	☐	Flow cytometry
☒	☐	MRI-based neuroimaging

Antibodies

Antibodies used	Rabbit polyclonal anti-beta2-microglobulin (ThermoFisher, cat. no. CL647-13511); rabbit polyclonal anti-Cdkn1c (ThermoFisher, cat. no. 23317 -1-AP); rabbit polyclonal anti-Cd63 antigen (ThermoFisher, cat. no. 25682-1-AP); mouse monoclonal anti-claudin-5, clone 4C3C2 (ThermoFisher, cat. no. 35-2500); rat monoclonal anti galectin-3, clone M3/38 (ThermoFisher, cat. no. 14-5301-82); rabbit polyclonal anti-LMX1B, serum BMO8 (Dev. Biol. 304, 701-712 (2007)); rabbit polyclonal anti-transgelin (Abcam, cat. no. ab14106)<br><br>Alexa Fluor 568-conjugated goat anti-mouse IgG (ThermoFisher, cat. no. A-11004); Alexa Fluor 568-conjugated goat anti-rabbit IgG (ThermoFisher, cat. no. A-11011); Alexa Fluor 633-conjugated goat anti-rabbit IgG (ThermoFisher, cat. no. A-21071)
Validation	Rabbit polyclonal anti-beta2-microglobulin was verified by immunofluorescence staining of A431 cells; rabbit polyclonal anti-Cdkn1c was verified by Western blot of HeLa cell extracts and by immunohistochemistry of human placenta tissue, it was also used in various publications; rabbit polyclonal anti-Cd63 antigen was verified by immunohistochemistry and immunofluorescence of e.g. human lymphoma tissue, human tonsillitis tissue and A431 cells, it was also used in various publications; mouse monoclonal anti-claudin-5, clone 4C3C2 was verified by Western blot of e.g. HUVEC extracts and by immunohistochemistry of human kidney tissue, it was also used in various publications; rat monoclonal anti galectin-3, clone M3/38 was verified by Western blot of e.g. MDA-MB-231 cells and by immunofluorescence of e.g. MDA-MB-231 cells, it was also used in various publications; rabbit polyclonal anti-LMX1B, serum BMO8 was generated by us and used as described in Dev. Biol. 304, 701-712 (2007); rabbit polyclonal anti-transgelin (Abcam, cat. no. ab14106) was verified by Western blot and immunofluorescence, it was also used in various publications.

Eukaryotic cell lines

Policy information about cell lines and Sex and Gender in Research

Cell line source(s)	HtTA-1 cells were provided by Hermann Bujard (Heidelberg). The human immortalized podocyte cell line was provided by Moin Saleem (Bristol).
Authentication	Cell lines were not further authenticated.
Mycoplasma contamination	All cell lines were tested negative for mycoplasma.
Commonly misidentified lines (See ICLAC register)	*Name any commonly misidentified cell lines used in the study and provide a rationale for their use.*

April 2023

Animals and other research organisms

Policy information about studies involving animals; ARRIVE guidelines recommended for reporting animal research, and Sex and Gender in Research

Laboratory animals	Mouse (C57BL/6 strain, age between embryonic day 18.5 and postnatal week 10)
Wild animals	The study did not involve wild animals.
Reporting on sex	Animal experiments investigating the efficacy of bortezomib were carried out separately with female and male mice involving between 12 and 18 mice of each sex and genotype.
Field-collected samples	The study did not involve samples collected from the field.
Ethics oversight	Animal experiments were approved by the Regierung von Unterfranken (Government of Lower Franconia) under the file number Az. 55.2.2 2532.2-1170.

Note that full information on the approval of the study protocol must also be provided in the manuscript.

Plants

Seed stocks	*Report on the source of all seed stocks or other plant material used. If applicable, state the seed stock centre and catalogue number. If plant specimens were collected from the field, describe the collection location, date and sampling procedures.*
Novel plant genotypes	*Describe the methods by which all novel plant genotypes were produced. This includes those generated by transgenic approaches, gene editing, chemical/radiation-based mutagenesis and hybridization. For transgenic lines, describe the transformation method, the number of independent lines analyzed and the generation upon which experiments were performed. For gene-edited lines, describe the editor used, the endogenous sequence targeted for editing, the targeting guide RNA sequence (if applicable) and how the editor was applied.*
Authentication	*Describe any authentication procedures for each seed stock used or novel genotype generated. Describe any experiments used to assess the effect of a mutation and, where applicable, how potential secondary effects (e.g. second site T-DNA insertions, mosiacism, off-target gene editing) were examined.*
