# Supplementary material for: Proteasome inhibition alleviates proteinuria in Lmx1b knock-in mice with dysfunctional LIM domains
Source: Nat Commun. 2026 Sep 11;17:9731. doi: 10.1038/s41467-026-77485-1 (PMC13562594; doi:10.1038/s41467-026-77485-1)
Supplement: Supplementary file 2 [file 41467_2026_77485_MOESM2_ESM.pdf]

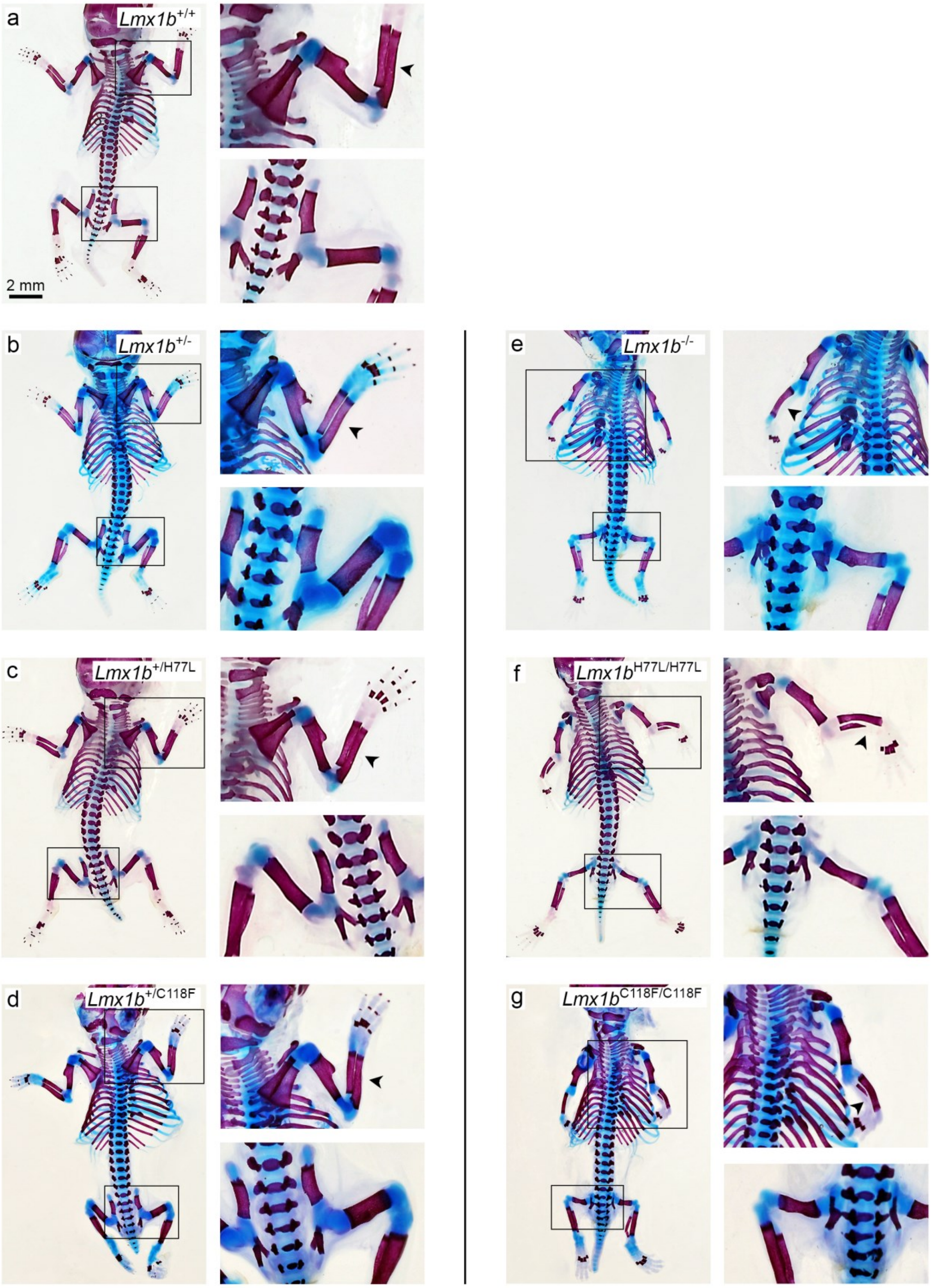

Suppl. Figure 1. Skeletal defects in *Lmx1b*$^{H77L/H77L}$ and *Lmx1b*$^{C118F/C118F}$ knock-in mice.

Mice were sacrificed on embryonic day 18.5. Similar to the situation in *Lmx1b*$^{-/-}$ mice (e),

skeletal staining with alcian blue and alizarin red demonstrates the absence of the distal end of the ulna or even the complete ulna (arrow heads) in *Lmx1b*$^{H77L/H77L}$ (f) and *Lmx1b*$^{C118F/C118F}$ (g) knock-in mice. Wild-type (a) and heterozygous (b-d) mice are shown for comparison.

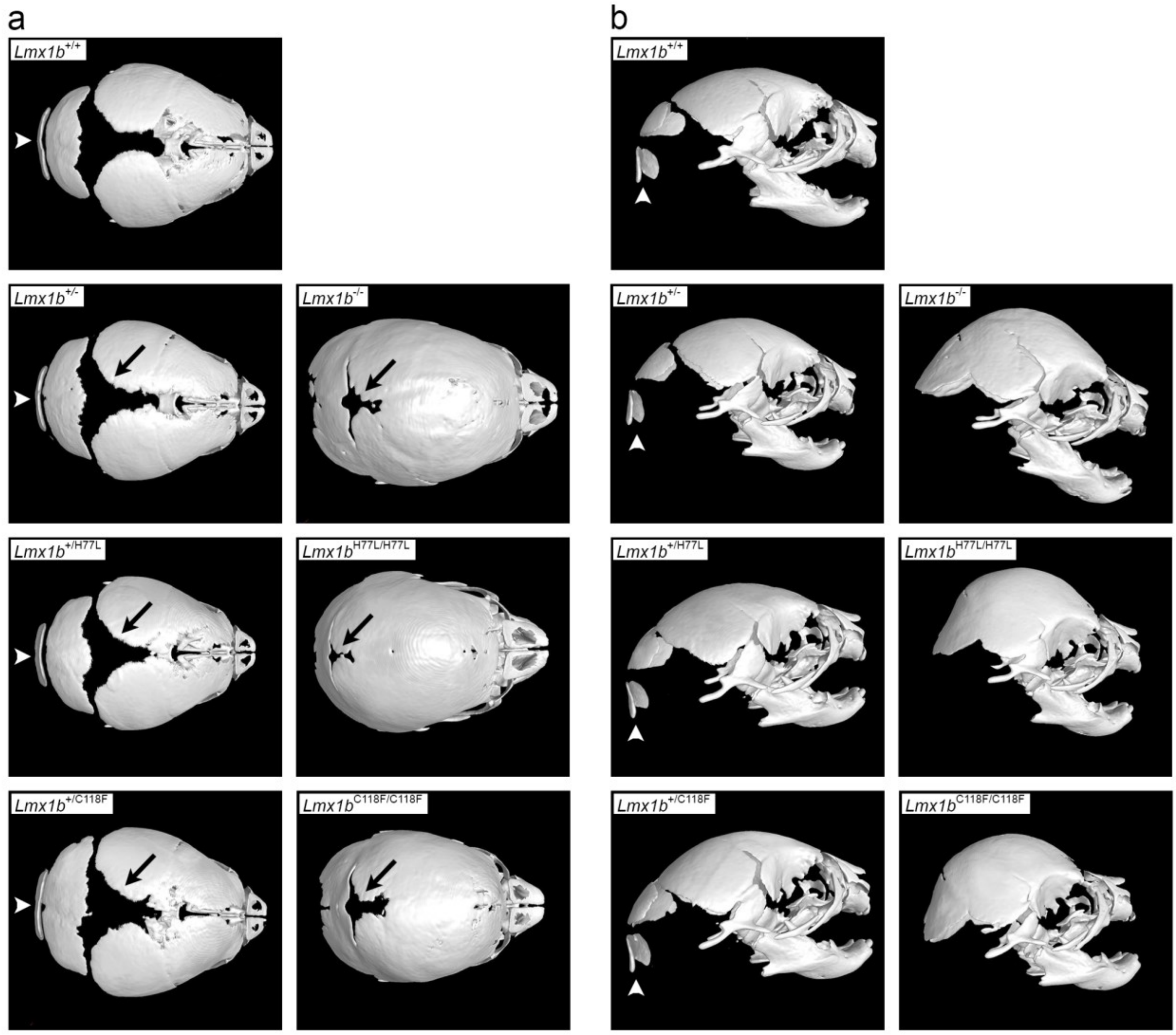

Suppl. Figure 2. Calvarial defects in *Lmx1b*$^{\mathbf{H77L/H77L}}$ and *Lmx1b*$^{\mathbf{C118F/C118F}}$ knock-in mice. Mice were sacrificed on embryonic day 18.5. The μCT scans demonstrate the absence of the supraoccipital bone (arrow heads), the smaller fontanelles (arrows) and the premature closure of the sutures in both homozygous knock-in lines. Wild-type, heterozygous and regular *Lmx1b* knock-out mice are shown for comparison. View from the top in a, side view in b.

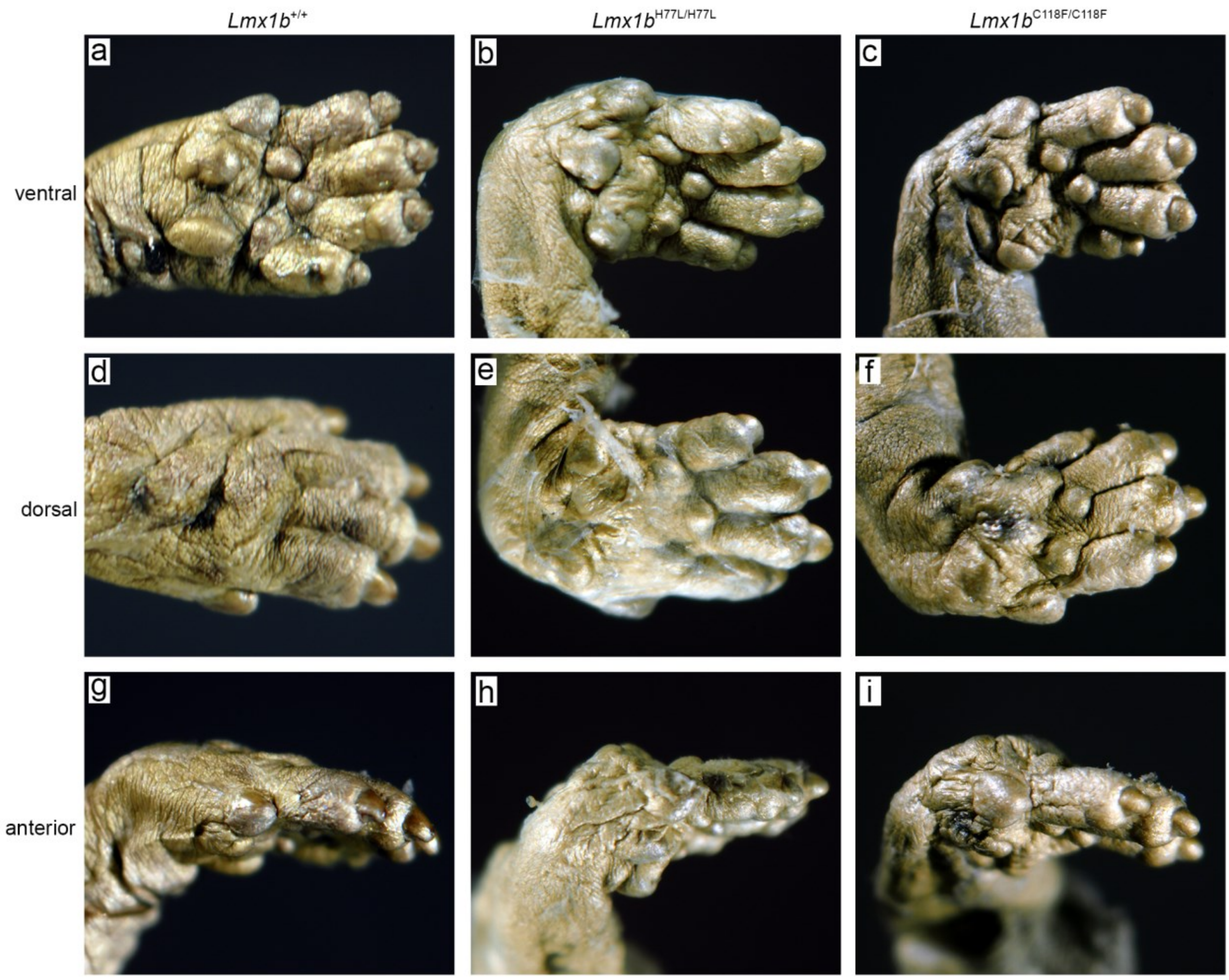

Suppl. Figure 3. Limb defects in *Lmx1b*$^{\mathrm{H77L/H77L}}$ and *Lmx1b*$^{\mathrm{C118F/C118F}}$ knock-in mice. Mice were sacrificed on embryonic day 18.5. Photographs show foot pads both on the ventral and the dorsal side in *Lmx1b*$^{\mathrm{H77L/H77L}}$ (b, e, h) and *Lmx1b*$^{\mathrm{C118F/C118F}}$ (c, f, i) knock-in mice. Wild-type mice are shown for comparison (a, d, g).

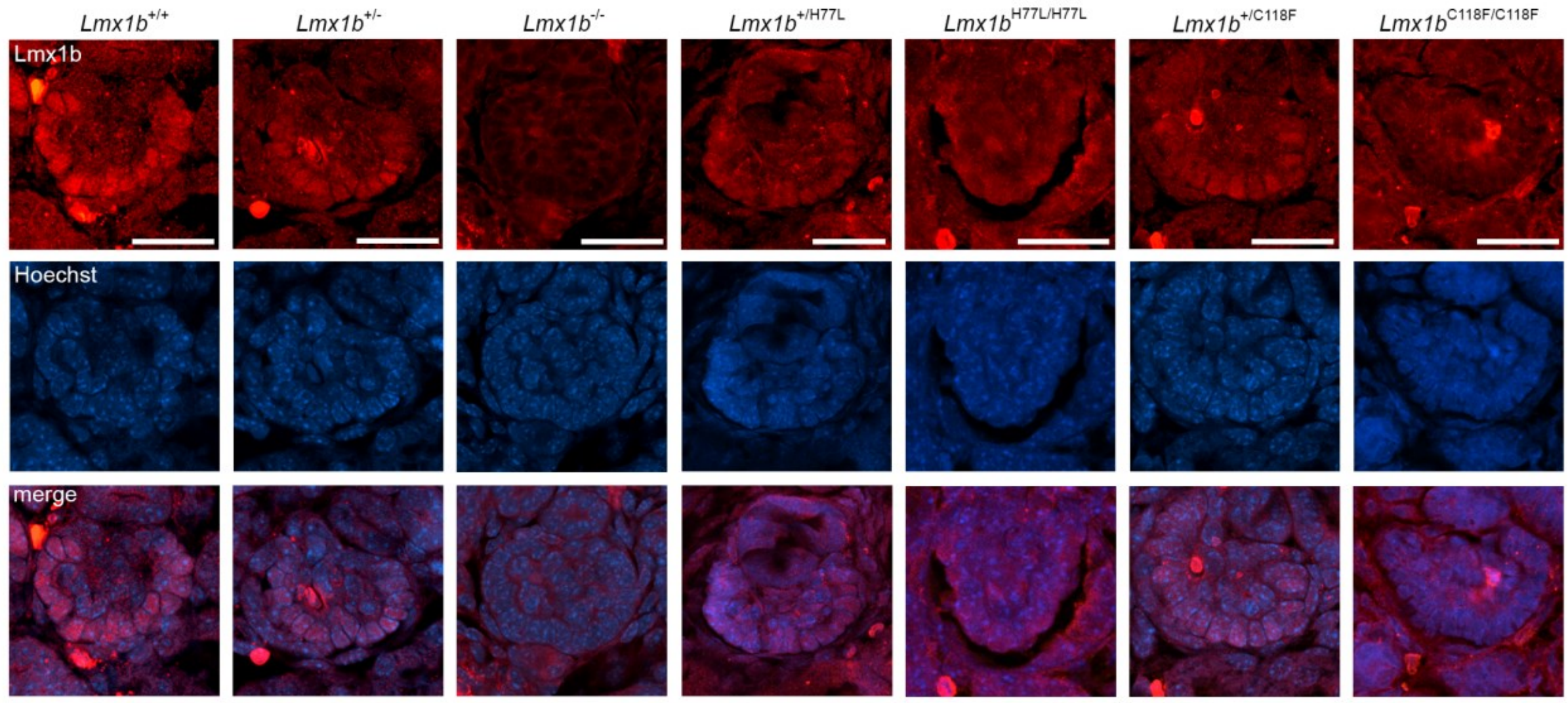

Suppl. Figure 4. The Lmx1b protein is absent in homozygous *Lmx1b* knock-in mice. Mice were sacrificed on embryonic day 18.5 and kidney sections stained for Lmx1b. The Lmx1b protein can be detected in podocytes of *Lmx1b*$^{+/+}$, *Lmx1b*$^{+/-}$, *Lmx1b*$^{+/\mathrm{H77L}}$ and *Lmx1b*$^{+/\mathrm{C118F}}$ but not of *Lmx1b*$^{-/-}$, *Lmx1b*$^{\mathrm{H77L/H77L}}$ and *Lmx1b*$^{\mathrm{C118F/C118F}}$ mice. Scale bars, 20 µm.

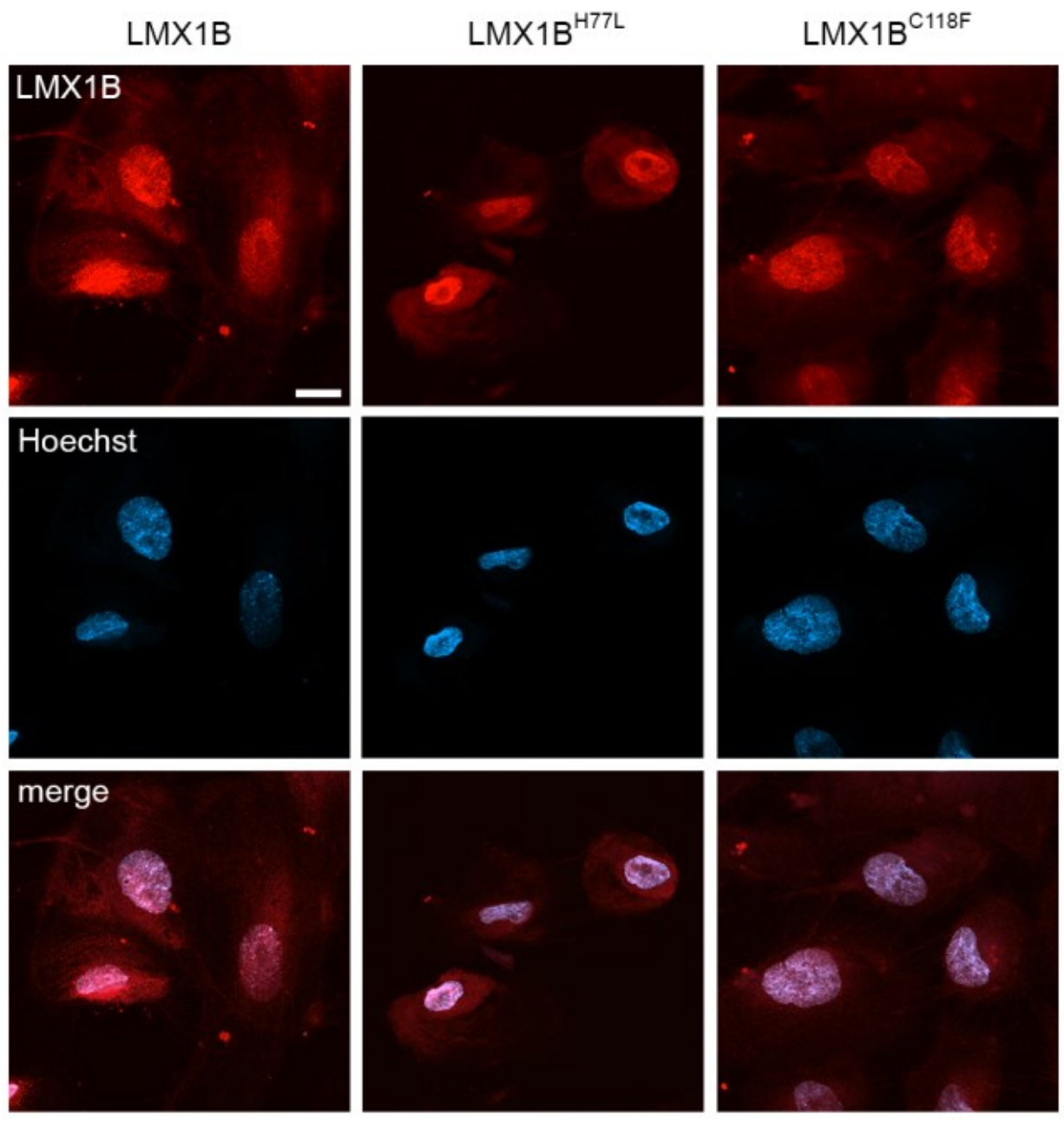

Suppl. Figure 5. Wild-type LMX1B as well as LMX1B^{H77L} and LMX1B^{C118F} mutant proteins are present in the nucleus. An immortalized human podocyte cell line was transduced with lentiviruses encoding human wild-type LMX1B, LMX1B^{H77L} and LMX1B^{C118F} proteins. Staining for LMX1B demonstrates that these proteins are sorted to the nucleus. Nuclei were counter-stained with Hoechst 33258 (Hoechst). Scale bar, 20 µm.

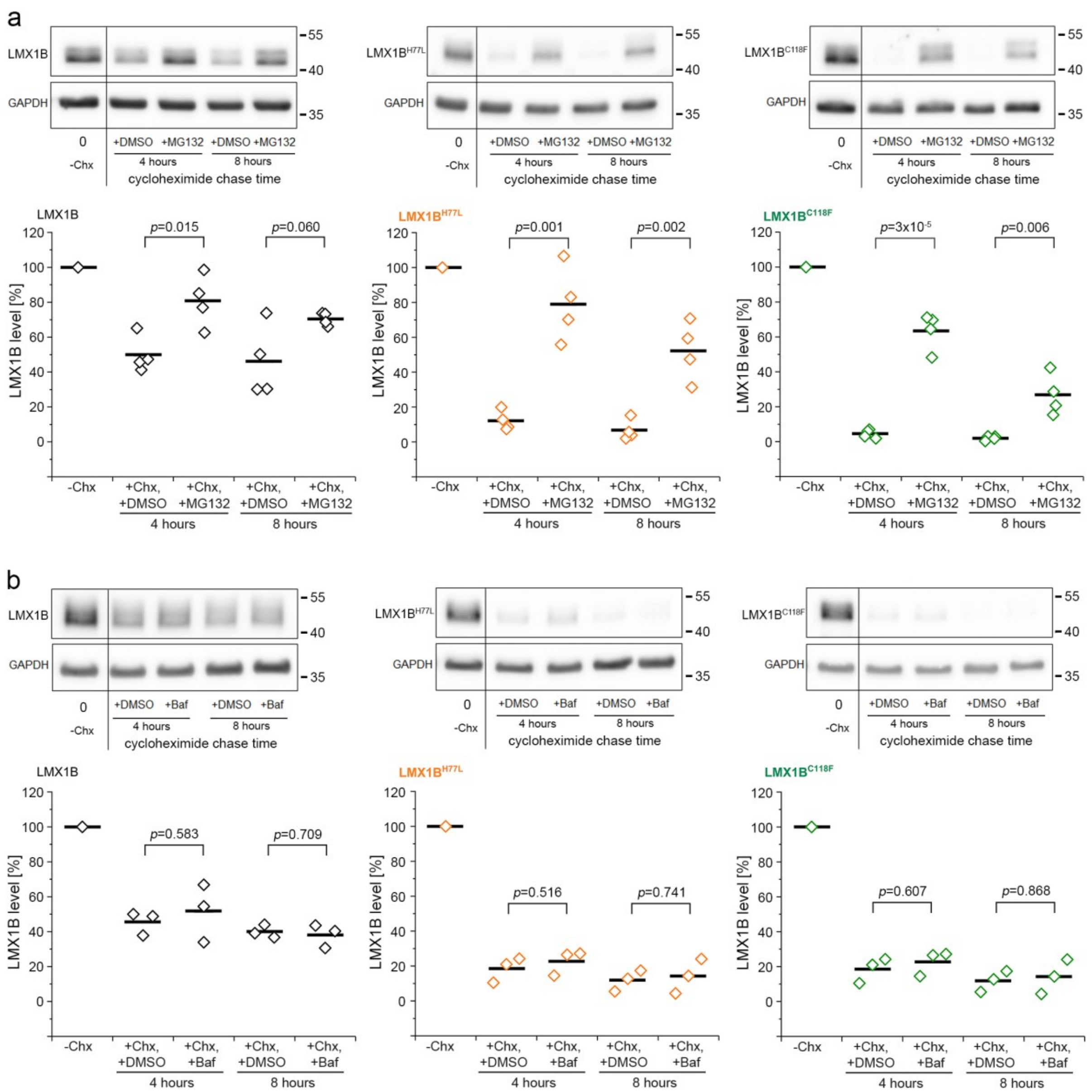

Suppl. Figure 6. The proteasomal inhibitor MG132 stabilizes the LMX1B^{H77L} and LMX1B^{C118F} mutant proteins, but the lysosomal inhibitor bafilomycin A_1 does not. Stably transfected HeLa cells inducibly producing human wild-type LMX1B, LMX1B^{H77L} and LMX1B^{C118F} proteins were used in the experiments. Six days after induction of LMX1B, cycloheximide (Chx) together with MG132 (a) or bafilomycin A_1 (b) (both at a final concentration of 10 µM) were simultaneously added to the culture medium to block protein synthesis and to inhibit the proteasome (MG132) or lysosomes (bafilomycin A_1), respectively (t = 0 hours), DMSO as solvent for MG132 and bafilomycin A_1 served as a negative control.

Cells were harvested at the indicated time points and 40 μg of total protein were subjected to Western blot analysis, the numbers on the right of each blot give the molecular mass in kDa. Glyceraldehyde-3-phosphate dehydrogenase (GAPDH) was used as a loading control. MG132 markedly prolongs the half-life of the LMX1B^{H77L} and LMX1B^{C118F} proteins but bafilomycin A_1 (Baf) does not. Shown are the results of four (a) and three (b) independent experiments, the horizontal lines represent the mean values. Statistical significance was determined by a two-sided unpaired Student's *t*-test. Source data are provided as a Source Data file.

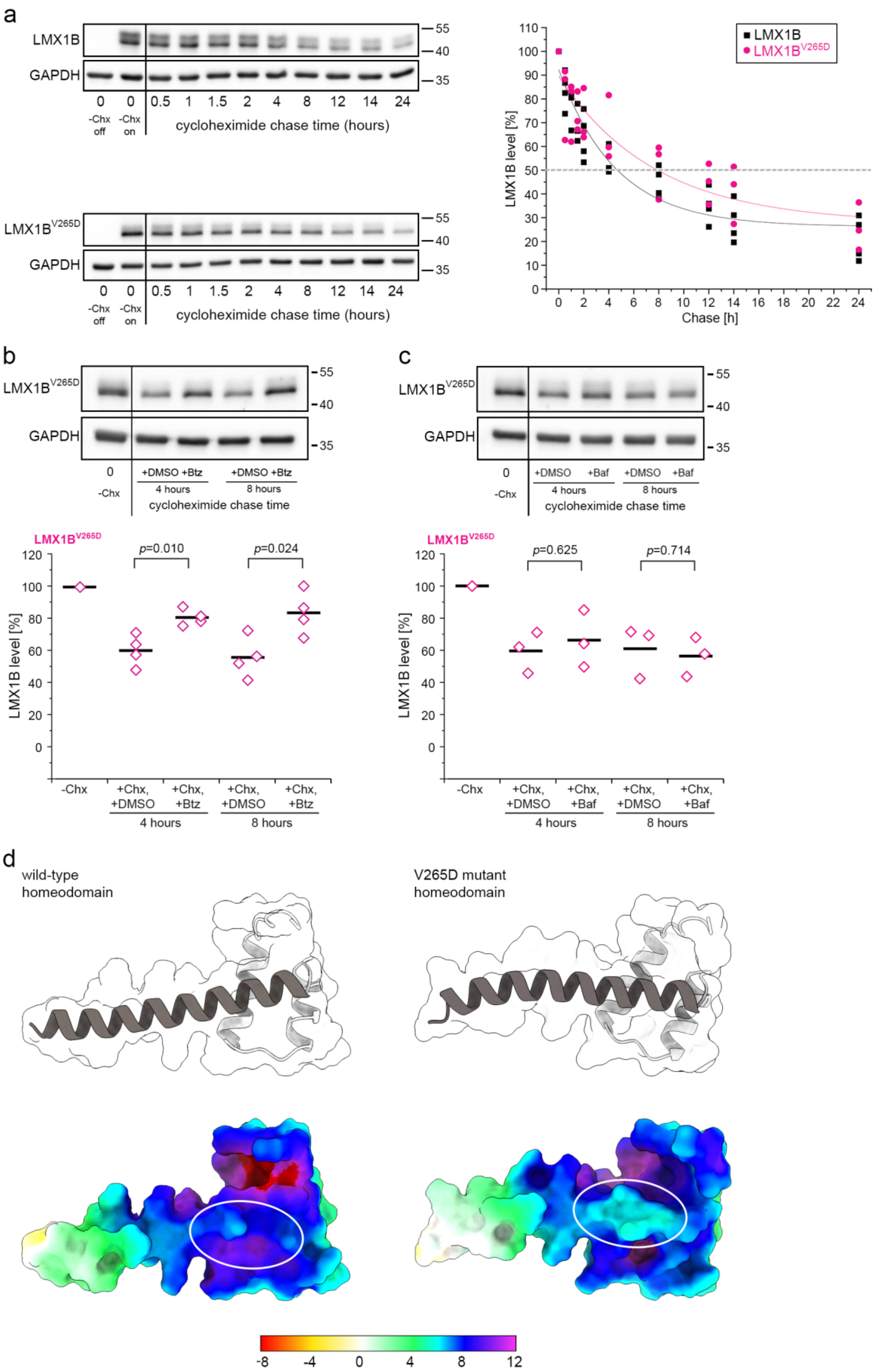
a
LMX1B
GAPDH
55
40
35
0 0 0.5 1 1.5 2 4 8 12 14 24
-Chx off
-Chx on
cycloheximide chase time (hours)
LMX1B^{V265D}
LMX1B
LMX1B^{V265D}
LMX1B level [%]
Chase [h]
b
+DMSO +Btz
4 hours
8 hours
cycloheximide chase time
p=0.010
p=0.024
c
+DMSO +Baf
p=0.625
p=0.714
-Chx
+Chx, +DMSO
+Chx, +Btz
+Chx, +Baf
d
wild-type homeodomain
V265D mutant homeodomain
-8 -4 0 4 8 12

Suppl. Figure 7. Stability of the LMX1B^{V265D} protein. Stably transfected HeLa cells inducibly producing human wild-type LMX1B and LMX1B^{V265D} proteins were used in the experiments. Cells were harvested at the indicated time points and 40 µg of total protein were subjected to Western blot analysis, the numbers on the right of each blot give the molecular mass in kDa. Glyceraldehyde-3-phosphate dehydrogenase (GAPDH) was used as a loading control. (a) Six days after induction of LMX1B (on), cycloheximide (Chx) was added into the culture medium to block protein synthesis (t = 0 hours), cells in which LMX1B synthesis was not induced (off) are shown as a negative control. Shown are the results of 4 independent experiments. (b) The experiment was carried out as described in (a), only that bortezomib (Btz) was added together with cycloheximide which prolonged the half-life of the LMX1B^{V265D} protein only moderately. The results of 4 independent experiments are presented, horizontal lines indicate the mean values. (c) The experiment was carried out as described in (a), only that bafilomycin A_1 (Baf) was added together with cycloheximide which did not prolong the half-life of the LMX1B^{V265D} protein. Shown are the results of 3 independent experiments, the horizontal lines represent the mean values. (d) Surface potential of the wild-type and the V265D mutant homeodomains of LMX1B. Shown are the ribbon diagrams of the homeodomain (the horizontal helix highlighted in dark grey indicates the DNA-binding helix) in the upper panel and three-dimensional models with the surface potential in the bottom panel. The replacement of valine by aspartate leads to a more negative surface potential (highlighted by the white oval) in the mutant protein (numbers of the color code give the surface potential in eV). Statistical significance was determined by a two-sided unpaired Student's t-test. Source data are provided as a Source Data file.

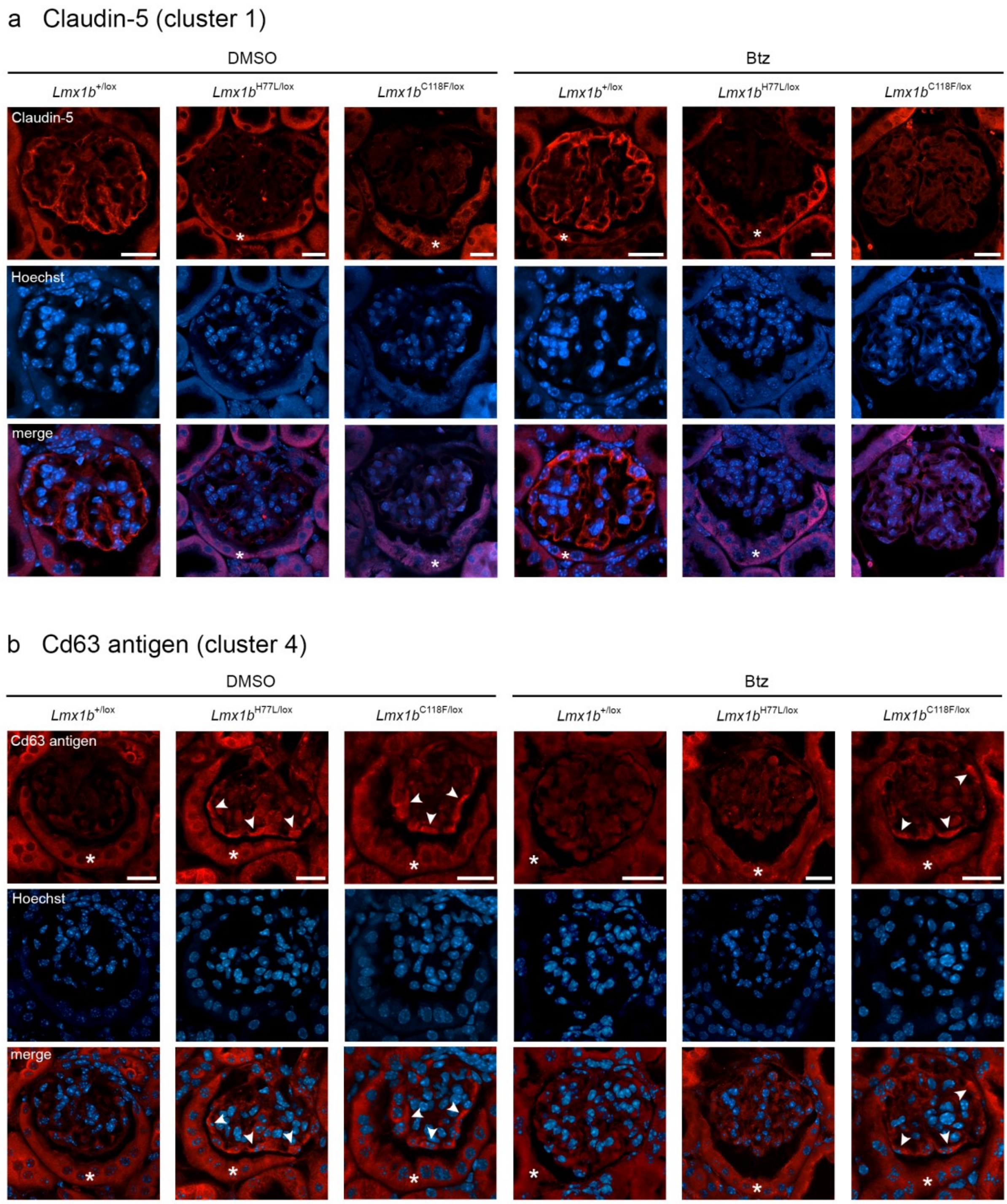

Suppl. Figure 8. Immunofluorescence analysis of proteins after treatment with DMSO and bortezomib. *Lmx1b*$^{+/lox}$, *Lmx1b*$^{H77L/lox}$ and *Lmx1b*$^{C118F/lox}$ mice with a podocyte-specific, doxycycline-inducible expression cassette for Cre recombinase (*Lmx1b*$^{+/lox}$ / *NPHS2*::rtTA / tetO::Cre mice; *Lmx1b*$^{H77L/lox}$ / *NPHS2*::rtTA / tetO::Cre mice; *Lmx1b*$^{C118F/lox}$ / *NPHS2*::rtTA / tetO::Cre mice) were analyzed. Mice were between 7-10 weeks of age. Induction of Cre

recombinase was started on day 0 by the administration of doxycycline in the drinking water together with the intraperitoneal injection of DMSO (solvent for bortezomib) or bortezomib every other day. On day 7, mice were perfusion-fixed and kidney sections were stained with the respective antibodies. Nuclei were counter-stained with Hoechst 33258. Three mice were analyzed for each genotype and treatment, a representative result in each case is shown. (a) Although claudin-5 is present in the podocytes of *Lmx1b*$^{+/lox}$ mice, it is undetectable in both knock-in lines independently of bortezomib treatment. (b) The Cd63 antigen is not detectable in the podocytes of *Lmx1b*$^{+/lox}$ mice but comes up in both DMSO-treated knock-in lines. This increase was prevented after bortezomib treatment in the *Lmx1b*$^{H77L/lox}$, but not the *Lmx1b*$^{C118F/lox}$, line. Arrow heads point to positive podocytes. Asterisks mark the proximal tubule of the urinary pole. *Lmx1b*$^{+/lox}$, *Lmx1b*$^{+/lox}$ / *NPHS2*::rtTA / tetO::Cre / mTmG mice; *Lmx1b*$^{H77L/lox}$, *Lmx1b*$^{H77L/lox}$ / *NPHS2*::rtTA / tetO::Cre / mTmG mice; *Lmx1b*$^{C118F/lox}$, *Lmx1b*$^{C118F/lox}$ / *NPHS2*::rtTA / tetO::Cre / mTmG mice. Scale bars, 20 µm.

Supplementary Table 1: Primary antibodies for immunofluorescence staining

Target	Host	Name	Dilution	Source
β2-microglobulin	Rabbit		1:400	ThermoFisher (cat. no. CL647-13511)
Cdkn1c	Rabbit		1:200	ThermoFisher (cat. no. 23317 -1-AP)
Cd63 antigen	Rabbit		1:600	ThermoFisher (cat. no. 25682-1-AP)
Claudin-5	Mouse	4C3C2	1:100	ThermoFisher (cat. no. 35-2500)
Galectin-3	Rat	M3/38	1:100	ThermoFisher (cat. no. 14-5301-82)
LMX1B	Rabbit	BMO8	1:800	*Dev. Biol.* **304**, 701-712 (2007)
Transgelin	Rabbit		1:200	Abcam (cat. no. ab14106)

Supplementary Table 2: Secondary antibodies for immunofluorescence staining

Target	Host	Fluorophore	Dilution	Source
Mouse IgG	Goat	Alexa Fluor 568	1:400	ThermoFisher (cat. no. A-11004)
Rabbit IgG	Goat	Alexa Fluor 568	1:400	ThermoFisher (cat. no. A-11011)
Rabbit IgG	Goat	Alexa Fluor 633	1:400	ThermoFisher (cat. no. A-21071)
Rat IgG	Goat	Alexa Fluor 488	1:400	ThermoFisher (cat. no. A-11006)

Supplementary Table 3: Oligonucleotides for qPCR (all murine mRNAs)

Target	Forward primer (5'-3')	Reverse primer (5'-3')	Length of PCR product [bp]
Filamin A	CTA CCT GTC TCA GTT TCC CAA G	GGG TCT CCA CAG TGA ATT CTG	149
Lamin	CAC CGC TCT CAT CAA CTC CA	GCT CTT CTC CAT CCT CGT CG	109
Lmx1b	AAA GAG CAA AGA TGA AGA AGA AGC TG	CGG CTT GAC AGA ACC TCT TG	93
Mustn1	GAG AGC TAC CAA CAG CGA G	ACT TGA TGT CCT GGT TCT TGG	148
Pak1	AAG ACA CTG GAA CCC TAA ACC	GAA ATC TCT GGT CGC TCC TTC	142
Serping1	CCT TTT CCC CAT TCA GCA TTG	TTG CTC TTG GTG CTG TCT C	73
