# Supplementary material for: Proteasome inhibition alleviates proteinuria in Lmx1b knock-in mice with dysfunctional LIM domains
Source: Nat Commun. 2026 Sep 11;17:9731. doi: 10.1038/s41467-026-77485-1 (PMC13562594; doi:10.1038/s41467-026-77485-1)
Supplement: Supplementary file 3 [file 41467_2026_77485_MOESM3_ESM.pdf]

nature portfolio

Peer Review File

Proteasome inhibition alleviates proteinuria in Lmx1b knock-in mice with dysfunctional LIM domains

Corresponding Author: Professor Ralph Witzgall

This file contains all reviewer reports in order by version, followed by all author rebuttals in order by version.

Version 0:

Reviewer comments:

Reviewer #1

(Remarks to the Author)
In this paper, Hermens et al. investigate the molecular mechanisms underlying nail-patella syndrome focusing on kidney pathology, which largely determines patient prognosis. The authors generate mouse models carrying human-like mutations in the LIM domains of LMX1B, demonstrating that these mutations lead to proteasomal degradation of the mutant proteins, thereby identifying a pathogenetic mechanism. They further show that the reduced stability of LMX1B is due to ubiquitin-mediated degradation, as proteasomal inhibitors can stabilize the mutant proteins and alleviate renal symptoms in mice. Using RNA sequencing of mutant and control podocytes, they reveal that LMX1B functions primarily as a transcriptional repressor, and that its two LIM domains differentially regulate distinct sets of target genes. The findings support further investigation of proteasomal inhibition as a targeted therapy for renal manifestations in NPS.
I believe this study offers valuable mechanistic insights into the pathogenesis of nail-patella syndrome and represents a meaningful contribution to the field. The work is particularly impressive for its sophisticated and precise use of mouse genetics, including the generation of multiple complex conditional mutations.
I would like to raise several points for the authors' consideration, which I hope may help strengthen the clarity the manuscript:
1. The authors replicate in mouse two mutations found in patients: LMX1BH77L and LMX1BC118F from reference 4. It would be helpful if they could elaborate on the rationale for selecting these specific mutations. I also suggest including any available clinical information on the disease manifestations, including renal involvement, in patients carrying these mutations.
2. The immunofluorescence images appear somewhat blurry. Could the authors provide higher-magnification images to better resolve the subcellular localization of the protein in both wild-type and mutant samples? Including DAPI staining would likely help clarify nuclear localization. Additionally, in Figure 7, since podocytes are expected to express both GFP and Lmx1b, shouldn't be overlap of signals? Is GFP localized to the membrane?
3. Please ensure that the complete genotype of each experimental mice is provided throughout the manuscript, at least at their first mention, both within the main text and in the figure legends.
4. Since with the authors tools the kidney phenotype can be induced when desired, I wonder whether they could test whether the phenotype could be reversed. Specifically, once podocyte foot processes have begun to be lost, could their structure be restored by stabilizing LMX1B protein? This question is highly significant for human health, as it would imply that treatment could be initiated after the onset of early renal symptoms/signs. The authors may want to consider allowing their mice to develop proteinuria and early kidney damage before initiating treatment with Bortezomib, to evaluate whether recovery is possible.
5. The RNA-seq analysis appears rather limited. Some of the mutant samples show considerable divergence from the others in the heat maps. Furthermore, none of the putative identified target genes have been validated, for example by in situ hybridization on tissue sections, and including such validation would be desirable.
Minor points:
• It would be helpful to include in the Introduction a brief discussion of the current hypothesis regarding the requirement of LDB1 co-factors for Lmx1b function.
• Some sentences are somewhat unclear and could benefit from rephrasing for clarity. For instance, in the Introduction: "...data from genetically manipulated mice in which parts of the Lmx1b gene were deleted..." , this sentence should make clear that the mutations result in loss of function. The specific details of the mutation are not essential in this context.
• Why was the patella not assessed, considering it is a superficial structure that is readily observable in skeletal stainings?
• Please specify the developmental stage (e.g., newborn, postnatal day X) at which each analysis was performed,

particularly in the figure legends.
• It would be useful to discuss the possibility that the BM08 antibody may not recognize the mutant protein.

Reviewer #2

(Remarks to the Author)
General Comments:
This manuscript evaluates the role of the two LIM domains of the Lmx1b transcription factor in podocyte development. The authors have done a significant amount of work to demonstrate that homozygote disruption of LIM-A or LIM-B impairs normal podocyte development and suggest that the presence of a single LIM domain is sufficient for podocyte development. They provide evidence that the mechanism underlying this altered development is the enhanced degradation of the LIM-mutant Lmx1b proteins. They further suggest that protease inhibitors may provide a therapeutic approach to LMX1B-mediated nephropathy and proteinuria. This is an important study, with novel findings, and provides meaningful insight into how some pathologic LMX1B variations cause haploinsufficiency in Nail-Patella Syndrome (NPS). Moreover, it provides a potential therapeutic intervention for a subpopulation of NPS patients with chronic renal dysfunction. This is an exciting therapeutic opportunity. However, we do have some concerns about the completeness of the LIM domain evaluation and the necessity of the genetic analysis performed on the mutant LIM domain.

Major concerns:
1) The authors suggest that the presence of a single LIM domain is sufficient for podocyte development/function, however this is based on the fact that one LIM domain is always present in their recovered protein model. It is unclear whether the genetic analysis of potential LIM targets (see below) identifies any target genes necessary for podocyte development or function. Did the authors consider a double knockout for both LIM domains to demonstrate whether the associated protein-protein interactions are required for podocyte development/maintenance? It seems logical that at least one is required, but there isn't data to support that assumption. Would loss of both LIM domains abate podocyte development even when protein degradation is blocked or are they dispensable for Lmx1b mediated podocyte development/function? Without this information it is unclear what roles LIM domains offer to podocyte development. They authors do however, provide compelling evidence that the mutant LIM domains trigger increased protein degradation, which decreases the levels of Lmx1b required for podocyte development. This is an unexpected and important finding.

2) The authors conduct a genetic analysis of mutant LIM-A Lmx1b and mutant LIM-B Lmx1b, comparing LIM-specific target genes to those in the heterozygote (Lmx1b+/-)(positive control), which is presumed to be similar to wildtype. While we applaud this innovative approach, it lacks a comparison with a Lmx1b construct that lacks LIM domains or with the Lmx1b-/- model (nonfunctional transcription factor) as a negative control. In other words, what gene targets are changed when both LIM domains are absent or when Lmx1b is non-functional. It appears that the authors have some of this data and have published on this in the past, as they note that Irf7 is upregulated in the conventional Lmx1b knock-out mice as well as the compound LIM heterozygotes. Moreover, it is not clear how any of these targets are linked to podocyte development/function. As the authors state in the discussion, these data are preliminary. Without these controls, and some link to podocyte development/function, what does the genetic analysis add to the story the authors are trying to convey? I don't believe it is central to, or supports, the main point which is that these LIM domain variants trigger degradation as a mechanism of Lmx1b haploinsufficiency. I believe that is a salient enough point. Thus, I would suggest either removing these studies or complete the analysis with negative controls and establish a link between the LIM domains and podocyte development.

3) In the discussion, the authors address the phenotypic differences between human and mice with pathologic heterozygotic alleles and indicate that there is a lack of an explanation for the discrepancy. What about a threshold level of Lmx1b being required to accomplish podocyte development? This threshold might be different in humans and mice, This would seem to be testable by using a sub-threshold level of bortezomib to stabilize some of the mutant proteins. In terms of musculoskeletal patterning, limb-specific cis-regulatory modules appear to amplify the levels of Lmx1b suggesting a threshold level is needed for musculoskeletal patterning. Similarly, the rapid degradation of the LIM domain mutants would reduce the levels of the transcription factor available to accomplish their function. Supporting this concept, the ulna (which is typically absent in the Lmx1b KO) in the LIM domain mutants was reduced but present in most of the examples shown suggesting partial, but incomplete function. In humans, both alleles appear to be necessary to maintain an adequate level of Lmx1b for podocyte function and haploinsufficiency puts podocyte function more at risk. So could not a species-specific difference in the required threshold of Lmx1b needed to accomplish its function explain their differences? To us this seems like an important consideration based on the data presented.

Minor Concerns:
1) The authors conducted protein degradation experiments thoroughly. However, we would appreciate clarification on why HeLa cells were chosen for these experiments instead of other cell lines, such as human embryonic kidney (HEK293) which would seem to be a more appropriate in vitro alternative.
2) In the first page of the results section a parenthetical statement seems incomplete "disease-causing variants of LMX1B (described as H54L and C95F in 4)" is the "in" needed or should it say in humans?
3) On the same page starting with the sentence that starts with "Contrary to our expectations … It is unclear whether you are referring to kidney or skeletal morphology. Please clarify for the reader and provide corresponding figure or citation. (I think one possible figure is Suppl Figure 2?
4) Please specify age of mice in the results or in methods section that was used for kidney staining
5) On the bottom of page six that carries over to page 7 the two sentences seem repetitive and could be condensed. Also as

noted in the previous comment, it would be helpful to know the age of the animals used for the kidney studies.
6) The first sentence of the section entitled “The LMX1BH77L and LMX1BC118F mutant proteins are unstable” is not clear. Could this be reworked for clarity?
7) It would be helpful for the readers to indicate that bafilomycin is an inhibitory of the process of autophagy.
8) The figure legend for Suppl Fig 2 indicates an arrow points to absence of a complete ulna, but no arrow is present, although there appears to be an absent ulna on the left of the C118F/C118F homozygote (g).

Reviewer #3

(Remarks to the Author)
I co-reviewed this manuscript with one of the reviewers who provided the listed reports. This is part of the Nature Communications initiative to facilitate training in peer review and to provide appropriate recognition for Early Career Researchers who co-review manuscripts.

Reviewer #4

(Remarks to the Author)
The manuscript „Proteasome inhibition alleviates proteinuria in Lmx1b knock-in mice with dysfunctional LIM domains“ submitted by Hermens et al. investigates the pathogenic mechanisms that lead to podocyte damage in respect to specific LMX1B gene mutations.
This is a beautiful and highly relevant study implying a multitude of transgenic mouse models, high quality histological and ultrastructural analyses, and carefully applied biochemical assays. The study is well written, would however profit from additional information here and there to make reading easier for the general public and the data more comprehensible. The manuscript would significantly profit, if the authors could show where the mutated and the subsequently through bortezomib stabilized Lmx1b protein localizes to subcellularly in podocytes? Predominantly to the nucleus like the wildtype protein, or are aggregates seen? Have the authors considered backcrossing their mice to a different background than BL6 to establish, whether heterozygosity results in a phenotype similar to patients? The BL6 background is very resistant to renal injury. Maybe this could be discussed. Bortezomib treatment is likely to also have prolonged effects on podocytes themselves, as these terminally differentiated cells are highly dependent on an intact degradative machinery. This should be added to the discussion. A validation of the presumable podocyte Lmx1b transcripts identified in the RNA sequencing screening in vivo needs to be shown in their transgenic mice.

Major points:
Fig.2: Please indicate the mouse age at which the morphological analyses were performed and are shown. This seems to be still the E18.5 embryos? Were glomeruli at the cortico-medullary junction (the presumably more mature glomeruli at that age) also chosen for EM? Please add an explanation for the choice of glomeruli for these investigations, as it is no trivial matter in developing kidneys. Were all developmental stages present in the different Lmx1b transgenic models, or was retardation of glomerular development visible?
Please show the Lmx1b protein expression separately from the context of bortezomib treatment (Fig. 7) as an additional panel in Fig. 2. It is unusual to refer to figure 7 after figure 2. Also specify the staining pattern (nuclear) of Lmx1b in your description.

Fig 3a. This is a beautiful experiment, especially as the GAPDH levels are maintained at a stable level. Why does the LMX1BH77L protein not exhibit an obvious double band like the wild type protein and the LMX1BC118F protein?
Fig 3b and c. The graphs are labeled wrong (4h / 4h instead of 4h / 8h) if they mirror the shown WB? Please also show the individual data points and indicate the statistical test used. Please indicate in the legend what that bortezomib is a pan proteasomal inhibitor and bafilomycin A a lysosomal inhibitor for the general reader. Information about the concentration and timing of inhibitor application should also be included.

Fig.4. Are the same samples shown on both membranes in panels a and b?

Fig. 4b. The graphs are labeled wrong (4h / 4h instead of 4h / 8h)? Please also show the individual data points and indicate the statistical test used. Please indicate in the legend what that bortezomib is a pan proteasomal inhibitor and bafilomycin A a lysosomal inhibitor for the general reader. Information about the concentration and timing of inhibitor application should also be included.

Fig. 6. Please include an experimental scheme that reflects the timing of the interventions performed in the mice as they remain largely unclear when reading the result text and figure legend. How old were they at the beginning of the experiment, for how long was the treatment performed? How long was the wash-out period? Was all residual Lmx1b protein lost at the time of proteinuria measurement? How soon after doxycycline administration did the mice start developing proteinuria? It seems the whole intervention (dox+bort) lasted only 7 days in total?

Fig. 8. For the general reader, a minimal labeling of the EM pictures is advisable.

Fig. 9. How old were the mice at the beginning of the experiment? Please show the PCA analyses of the datasets. Were podocyte-specific transcripts identified?
Please rephrase: “When bortezomib was administered to Lmx1b+/lox mice every other day, no obvious difference was detected to the DMSO-treated Lmx1b+/lox animals (Fig. 10a), corroborating the notion that bortezomib showed no

nephrotoxic effect“ as the presented data are not suitable for the assessment of nephrotoxicity.

Fig. 10. Please show qPCR validation of the putative Lmx1b specific genes and validate an effect of their transcript regulation for the respective protein levels in podocytes (WB or IF).

Suppl. Fig 4: Please show the individual data points.

Suppl. Fig. 5: The graphs are labeled wrong (4h / 4h instead of 4h / 8h)? Please also show the individual data points and indicate the statistical test used. Please indicate in the legend what that MG132. Information about the concentration and timing of inhibitor application should also be included.

Minor points:
Page 3 Introduction: Please clarify at the beginning of this text section, which discrepancies are being addressed. Between constitutive and podocyte-specific KO or between the patient mutations and the murine KO models (constitutive or inducible)?

Figure legend 1: Please write out „resp.“

Suppl. Fig. 1: Please add a scale bar.

Figure legend 4: Sentence unclear: „Only in the case of the two mutants, but not the wild-type, LMX1B proteins an additional smear can be detected in the presence of the HA-tagged ubiquitin chains.“

Version 1:

Reviewer comments:

Reviewer #1

(Remarks to the Author)

Reviewer #4

(Remarks to the Author)
The authors have sufficiently addressed and answered my concerns. The manuscript was revised appropriately.

Open Access This Peer Review File is licensed under a Creative Commons Attribution 4.0 International License, which permits use, sharing, adaptation, distribution and reproduction in any medium or format, as long as you give appropriate credit to the original author(s) and the source, provide a link to the Creative Commons license, and indicate if changes were

made.
In cases where reviewers are anonymous, credit should be given to 'Anonymous Referee' and the source.
The images or other third party material in this Peer Review File are included in the article's Creative Commons license, unless indicated otherwise in a credit line to the material. If material is not included in the article's Creative Commons license and your intended use is not permitted by statutory regulation or exceeds the permitted use, you will need to obtain permission directly from the copyright holder.
To view a copy of this license, visit https://creativecommons.org/licenses/by/4.0/

Reply to the comments of reviewer #1

(i) In this paper, Hermens et al. investigate the molecular mechanisms underlying nail-patella syndrome focusing on kidney pathology, which largely determines patient prognosis. The authors generate mouse models carrying human-like mutations in the LIM domains of LMX1B, demonstrating that these mutations lead to proteasomal degradation of the mutant proteins, thereby identifying a pathogenetic mechanism. They further show that the reduced stability of LMX1B is due to ubiquitin-mediated degradation, as proteasomal inhibitors can stabilize the mutant proteins and alleviate renal symptoms in mice. Using RNA sequencing of mutant and control podocytes, they reveal that LMX1B functions primarily as a transcriptional repressor, and that its two LIM domains differentially regulate distinct sets of target genes. The findings support further investigation of proteasomal inhibition as a targeted therapy for renal manifestations in NPS. I believe this study offers valuable mechanistic insights into the pathogenesis of nail-patella syndrome and represents a meaningful contribution to the field. The work is particularly impressive for its sophisticated and precise use of mouse genetics, including the generation of multiple complex conditional mutations.

We are very grateful for the positive overall assessment of the reviewer.

(Major point 1) The authors replicate in mouse two mutations found in patients: LMX1BH77L and LMX1BC118F from reference 4. It would be helpful if they could elaborate on the rationale for selecting these specific mutations. I also suggest including any available clinical information on the disease manifestations, including renal involvement, in patients carrying these mutations.

LIM domains contain 8 zinc-coordinating amino acids which are cysteine, histidine and aspartic acid. Missense mutations in patients with nail-patella syndrome have been described for any of those amino acids. Regrettably it has not been possible so far to establish a genotype-phenotype correlation with respect to the type and position of the mutations and therefore there were no clinical data available on which we could base our choice. For practical reasons we focused on one zinc-coordinating amino acid each in the LIM-A and LIM-B domains.

(Major point 2) The immunofluorescence images appear somewhat blurry. Could the authors provide higher-magnification images to better resolve the subcellular localization of the protein in both wild-type and mutant samples? Including DAPI staining would likely help clarify nuclear localization. Additionally, in Figure 7, since podocytes are expected to express both GFP and Lmx1b, shouldn't be overlap of signals? Is GFP localized to the membrane?

We now provide new confocal images on the location of the wild-type and mutant LMX1B proteins in renal sections (Fig. 7). Nuclei were stained with Hoechst 33258 (instead of DAPI). Furthermore we have determined the subcellular distribution of the various LMX1B proteins in transduced podocyte cell lines and detect a nuclear location for both wild-type LMX1B and the two mutant proteins (suppl. Fig. 6). Therefore the mutations do not affect the subcellular distribution of LMX1B.

Regarding Figure 7, the GFP variant used in the mice is sorted to the plasma membrane, therefore no overlap is expected between the GFP and Lmx1b signals.

(Major point 3) Please ensure that the complete genotype of each experimental mice is provided throughout the manuscript, at least at their first mention, both within the main text and in the figure legends.

This has been done.

(Major point 4) Since with the authors tools the kidney phenotype can be induced when desired, I wonder whether they could test whether the phenotype could be reversed. Specifically, once podocyte foot processes have begun to be lost, could their structure be restored by stabilizing LMX1B protein? This question is highly significant for human health, as it would imply that treatment could be initiated after the onset of early renal symptoms/signs. The authors may want to consider allowing their mice to develop proteinuria and early kidney damage before initiating treatment with Bortezomib, to evaluate whether recovery is possible.

The reviewer asks a very legitimate and intriguing question, as a matter of fact we have also thought about this experiment. However, in order to carry out such a study we would have to file a new animal protocol with the local authorities. The frustrating experience shows that it takes in the order of nine months and longer to obtain approval (and before we could even begin with our experiments) which we think would be beyond the scope of the current manuscript.

(Major point 5) The RNA-seq analysis appears rather limited. Some of the mutant samples show considerable divergence from the others in the heat maps. Furthermore, none of the putative identified target genes have been validated, for example by in situ hybridization on tissue sections, and including such validation would be desirable.

Immunofluorescence stainings of selected proteins representing the four different clusters were performed with suitable antibodies, the expression of additional genes was analyzed by qPCR. By those two approaches, we have mostly been able to validate the expression patterns predicted from the cluster analysis. Accordingly, the respective genes are now pointed out in the volcano blot in Fig. 9 and previous Fig. 10b has been replaced by the immunofluorescence stainings (Fig. 11) and the qPCR data (Fig. 12).

(Minor point 1) It would be helpful to include in the Introduction a brief discussion of the current hypothesis regarding the requirement of LDB1 co-factors for Lmx1b function.

This has been done.

(Minor point 2) Some sentences are somewhat unclear and could benefit from rephrasing for clarity. For instance, in the Introduction: "...data from genetically manipulated mice in which parts of the Lmx1b gene were deleted..." , this sentence should make clear that the mutations result in loss of function. The specific details of the mutation are not essential in this context.

We consider it helpful to mention the various animal models in this context. For example, the conventional *Lmx1b* knock-out mice already die on the day of birth and therefore are difficult

to use for further analysis of the podocyte phenotype. Hopefully the reviewer therefore agrees that we keep those details.

(Minor point 3) Why was the patella not assessed, considering it is a superficial structure that is readily observable in skeletal stainings?

In our experience the patellae are not readily observed in skeletal staining even of wild-type mouse embryos on gestational day 18. Therefore we decided not to assess the presence and absence of patellae.

(Minor point 4) Please specify the developmental stage (e.g., newborn, postnatal day X) at which each analysis was performed, particularly in the figure legends.

This has been done.

(Minor point 5) It would be useful to discuss the possibility that the BM08 antibody may not recognize the mutant protein.

We have performed Western blots of myc-epitope-tagged wild-type and mutant LMX1B proteins to make sure that the anti-LMX1B antibody does not preferentially detect wild-type LMX1B. The anti-myc-epitope antibody was used as an internal control to which the signal obtained with the anti-LMX1B antibody was normalized. In two independent experiments no difference in the binding of the anti-LMX1B antibody between wild-type and mutant proteins was detectable. Compared to the signal intensity of wild-type LMX1B, which was set at 100%, we observed 88% and 111% signal intensity, respectively, for LMX1B^{H77L}, and 128% and 99% signal intensity, respectively, for LMX1B^{C118F}.

Reply to the comments of reviewer #2

(i) This manuscript evaluates the role of the two LIM domains of the Lmx1b transcription factor in podocyte development. The authors have done a significant amount of work to demonstrate that homozygote disruption of LIM-A or LIM-B impairs normal podocyte development and suggest that the presence of a single LIM domain is sufficient for podocyte development. They provide evidence that the mechanism underlying this altered development is the enhanced degradation of the LIM-mutant Lmx1b proteins. They further suggest that protease inhibitors may provide a therapeutic approach to LMX1B-mediated nephropathy and proteinuria. This is an important study, with novel findings, and provides meaningful insight into how some pathologic LMX1B variations cause haploinsufficiency in Nail-Patella Syndrome (NPS). Moreover, it provides a potential therapeutic intervention for a subpopulation of NPS patients with chronic renal dysfunction. This is an exciting therapeutic opportunity. However, we do have some concerns about the completeness of the LIM domain evaluation and the necessity of the genetic analysis performed on the mutant LIM domain.

We are very grateful that the reviewer thinks highly of our manuscript.

(Major point 1) The authors suggest that the presence of a single LIM domain is sufficient for podocyte development/function, however this is based on the fact that one LIM domain is always present in their recovered protein model. It is unclear whether the genetic analysis of potential LIM targets (see below) identifies any target genes necessary for podocyte development or function. Did the authors consider a double knockout for both LIM domains to demonstrate whether the associated protein-protein interactions are required for podocyte development/maintenance? It seems logical that at least one is required, but there isn't data to support that assumption. Would loss of both LIM domains abate podocyte development even when protein degradation is blocked or are they dispensable for Lmx1b mediated podocyte development/function? Without this information it is unclear what roles LIM domains offer to podocyte development. They authors do however, provide compelling evidence that the mutant LIM domains trigger increased protein degradation, which decreases the levels of Lmx1b required for podocyte development. This is an unexpected and important finding.

The reviewer raises a very interesting point. However, the two exons encoding the LIM-A and LIM-B domains lie so close to each other that it is virtually impossible to obtain mice carrying mutations in both LIM domains by simply crossing both lines with each other. We would have to use one of the two knock-in lines and introduce the desired point mutation in the other exon, establish a colony and carry out the necessary experiments. Realistically speaking we are talking about one and a half to two years altogether, which we consider beyond the scope of the present manuscript.

(Major point 2) The authors conduct a genetic analysis of mutant LIM-A Lmx1b and mutant LIM-B Lmx1b, comparing LIM-specific target genes to those in the heterozygote (Lmx1b+/-)(positive control), which is presumed to be similar to wildtype. While we applaud this innovative approach, it lacks a comparison with a Lmx1b construct that lacks LIM domains or with the Lmx1b-/- model (nonfunctional transcription factor) as a negative control. In other words, what gene targets are changed when both LIM domains are absent or when Lmx1b is non-functional. It appears that the authors have some of this data and have published on this in the past, as they note that Irf7 is upregulated in the conventional Lmx1b knock-out mice as well as the compound LIM heterozygotes. Moreover, it is not clear how any of these targets are linked to podocyte development/function. As the authors state in the discussion, these data are preliminary. Without these controls, and some link to podocyte development/function, what does the genetic analysis add to the story the authors are trying to convey? I don't believe it is central to, or supports, the main point which is that these LIM domain variants trigger degradation as a mechanism of Lmx1b haploinsufficiency. I believe that is a salient enough point. Thus, I would suggest either removing these studies or complete the analysis with negative controls and establish a link between the LIM domains and podocyte development.

The reviewer wonders what the RNA sequencing analysis adds to our story. As already discussed in our manuscript, we would like to emphasize that one motivation of our study was to find out whether the two LIM domains of Lmx1b serve distinct functions, i.e. whether one of them is more important than the other in podocytes. Due to the fact that the mutant proteins are unstable and homozygous knock-in mice present with a lethal phenotype similar to the conventional *Lmx1b* knock-out mice, we consider podocytes of induced compound heterozygous knock-in mice (*Lmx1b*$^{H77L/lox}$ and *Lmx1b*$^{C118F/lox}$) treated with DMSO a reasonable control for the respective comparisons. We acknowledge that we did not obtain a

straightforward answer to this question but nevertheless we still believe that it was acceptable to resort to RNA sequencing of freshly isolated podocytes. Here indeed we have obtained evidence that the two LIM domains, at least to some extent, act in a contextual fashion because different genes are up- and down-regulated depending on what LIM domain is mutated. Additionally, our GO analysis in Figure 9 provides an overview of the functions of the putative target genes and revealed several intriguing associations such as 'cell adhesion' and 'external encapsulating structures'. When appropriate and applicable, we now also point out the relevance of the respective proteins for podocyte function. Furthermore, as also requested by the other reviewers, we have verified the expression of several of the genes identified by RNA sequencing by immunostaining and by qPCR. Accordingly, the respective genes are now pointed out in the volcano blot in Fig. 9 and previous Fig. 10b has been replaced by the immunofluorescence stainings (Fig. 11) and the qPCR data (Fig. 12).

Therefore we believe that this is an important piece of information which rounds off our story, and would appreciate greatly if this reviewer consents to keeping the data in the manuscript.

(Major point 3) In the discussion, the authors address the phenotypic differences between human and mice with pathologic heterozygotic alleles and indicate that there is a lack of an explanation for the discrepancy. What about a threshold level of Lmx1b being required to accomplish podocyte development? This threshold might be different in humans and mice, This would seem to be testable by using a sub-threshold level of bortezomib to stabilize some of the mutant proteins. In terms of musculoskeletal patterning, limb-specific cis-regulatory modules appear to amplify the levels of Lmx1b suggesting a threshold level is needed for musculoskeletal patterning. Similarly, the rapid degradation of the LIM domain mutants would reduce the levels of the transcription factor available to accomplish their function. Supporting this concept, the ulna (which is typically absent in the Lmx1b KO) in the LIM domain mutants was reduced but present in most of the examples shown suggesting partial, but incomplete function. In humans, both alleles appear to be necessary to maintain an adequate level of Lmx1b for podocyte function and haploinsufficiency puts podocyte function more at risk. So could not a species-specific difference in the required threshold of Lmx1b needed to accomplish its function explain their differences? To us this seems like an important consideration based on the data presented.

The explanation by the reviewer that differences in the LMX1B/Lmx1b levels between human and mouse underlie the distinct symptoms in the two species is well taken. To determine a threshold level at which Lmx1b prevents proteinuria in the mouse would require a titration experiment with bortezomib. Even if we knew at what dosage bortezomib would still show a therapeutic effect in the mouse, it would not be possible to determine LMX1B levels in human kidneys and compare the two species to each other. Furthermore, as already elaborated in an answer to one of the questions from reviewer #1, it takes at least 9 months in Germany to get approval for a new animal protocol. With all due respect to the reviewer, we therefore believe that such an experiment is beyond the scope of the present manuscript.

(Minor point 1) The authors conducted protein degradation experiments thoroughly. However, we would appreciate clarification on why HeLa cells were chosen for these experiments instead of other cell lines, such as human embryonic kidney (HEK293) which would seem to be a more appropriate in vitro alternative.

In our experience LMX1B has an inhibitory effect on the growth of cell lines, therefore we had to establish an inducible cell line and could not use a constitutive expression system. We have obtained good results with inducible HeLa cells which is why we resorted to this cell line and not HEK 293 cells.

(Minor point 2) In the first page of the results section a parenthetical statement seems incomplete "disease-causing variants of LMX1B (described as H54L and C95F in 4)" is the "in" needed or should it say in humans?

The „in" refers to the reference, we have corrected that accordingly.

(Minor point 3) On the same page starting with the sentence that starts with "Contrary to our expectations ... It is unclear whether you are referring to kidney or skeletal morphology. Please clarify for the reader and provide corresponding figure or citation. (I think one possible figure is Suppl Figure 2?

We now refer to supplementary figures 1 and 2 (previous supplementary figures 2 and 3).

(Minor point 4) Please specify age of mice in the results or in methods section that was used for kidney staining.

This has been done, the information is now present in the legends.

(Minor point 5) On the bottom of page six that carries over to page 7 the two sentences seem repetitive and could be condensed. Also as noted in the previous comment, it would be helpful to know the age of the animals used for the kidney studies.

In the first sentence we are comparing the <u>heterozygous and homozygous</u> knock-in mice, whereas in the second sentence we are comparing the <u>knock-in and knock-out</u> mice. We have rephrased these two sentences to make our point easier to understand.

The age of the mice is now mentioned.

(Minor point 6) The first sentence of the section entitled "The LMX1BH77L and LMX1BC118F mutant proteins are unstable" is not clear. Could this be reworked for clarity?

This has been done.

(Minor point 7) It would be helpful for the readers to indicate that bafilomycin is an inhibitory of the process of autophagy.

This has been done.

(Minor point 8) The figure legend for Suppl Fig 2 indicates an arrow points to absence of a complete ulna, but no arrow is present, although there appears to be an absent ulna on the left of the C118F/C118F homozygote (g).

With all due respect to the reviewer, but there was an arrow head present in panel g already (now suppl. Fig. 1). In addition, we now point out the ulna in all panels.

Reply to the comments of reviewer #4

(i) The manuscript „Proteasome inhibition alleviates proteinuria in Lmx1b knock-in mice with dysfunctional LIM domains“ submitted by Hermens et al. investigates the pathogenic mechanisms that lead to podocyte damage in respect to specific LMX1B gene mutations. This is a beautiful and highly relevant study implying a multitude of transgenic mouse models, high quality histological and ultrastructural analyses, and carefully applied biochemical assays. The study is well written, would however profit from additional information here and there to make reading easier for the general public and the data more comprehensible.

We are very grateful for the reviewer's high opinion of our manuscript.

(ii) The manuscript would significantly profit, if the authors could show where the mutated and the subsequently through bortezomib stabilized Lmx1b protein localizes to subcellularly in podocytes? Predominantly to the nucleus like the wildtype protein, or are aggregates seen?

We have localized the wild-type and mutant LMX1B proteins to the nucleus both on kidney sections (Fig. 7) and in stably transduced immortalized podocyte cell lines (suppl. Fig. 6). Therefore the mutated proteins reach their physiological location, i.e. the nucleus, and are positioned to act on the relevant target genes. This is now mentioned in the manuscript.

(iii) Have the authors considered backcrossing their mice to a different background than BL6 to establish, whether heterozygosity results in a phenotype similar to patients? The BL6 background is very resistant to renal injury. Maybe this could be discussed.

The reviewer brings up a very relevant topic, mice with a C57BL/6 genetic background are less sensitive to various kinds of renal injury than other backgrounds. We now mention this fact in the discussion. At the same time we consider it beyond the scope of the present manuscript to examine other strains because we are not only dealing with very complex crosses with various *Lmx1b* alleles and additional expression cassettes such as *NPHS2*:rtTA and tetO:Cre, but a pure

genetic background is generally only accepted after 10 back crosses which amounts to two and a half to three years of breeding.

(iv) Bortezomib treatment is likely to also have prolonged effects on podocytes themselves, as these terminally differentiated cells are highly dependent on an intact degradative machinery. This should be added to the discussion.

This has been done.

(v) A validation of the presumable podocyte Lmx1b transcripts identified in the RNA sequencing screening in vivo needs to be shown in their transgenic mice.

Immunofluorescence stainings of selected proteins representing the four different clusters were performed with suitable antibodies, the expression of additional genes was analyzed by qPCR. By those two approaches, we have mostly been able to validate the expression patterns predicted from the cluster analysis.

(Major point 1) Fig.2: Please indicate the mouse age at which the morphological analyses were performed and are shown. This seems to be still the E18.5 embryos? Were glomeruli at the cortico-medullary junction (the presumably more mature glomeruli at that age) also chosen for EM? Please add an explanation for the choice of glomeruli for these investigations, as it is no trivial matter in developing kidneys. Were all developmental stages present in the different Lmx1b transgenic models, or was retardation of glomerular development visible?

We have added the age of the animals to the legends (yes, the pictures are from E18.5 kidneys as now mentioned in the figure legend).

The reviewer raises an extremely important point because because the comparison of superficial glomeruli, which lack behind in development, and of glomeruli deeper in the cortex, which have advanced furthest in development, would be like comparing apples and oranges. We want

to assure the reviewer that we took great caution to pick glomeruli at the cortico-medullary border in the various kidneys.

We observed a retardation of glomerular development in the two *Lmx1b* knock-in lines in the sense that podocytes did not mature. Accordingly the glomerular tuft, whose development depends on podocytes, also showed a lack of branching.

(Major point 2) Please show the Lmx1b protein expression separately from the context of bortezomib treatment (Fig. 7) as an additional panel in Fig. 2. It is unusual to refer to figure 7 after figure 2. Also specify the staining pattern (nuclear) of Lmx1b in your description.

We have now added a new supplementary Figure 5 to demonstrate the absence of the mutant Lmx1b proteins in homozygous *Lmx1b*$^{H77L/H77L}$ and *Lmx1b*$^{C118F/C118F}$ mice. The nuclear location of the various LMX1B/Lmx1b proteins is demonstrated in Figure 7 and supplementary Figure 6.

(Major point 3) Fig 3a. This is a beautiful experiment, especially as the GAPDH levels are maintained at a stable level. Why does the LMX1BH77L protein not exhibit an obvious double band like the wild type protein and the LMX1BC118F protein? Fig 3b and c. The graphs are labeled wrong (4h / 4h instead of 4h / 8h) if they mirror the shown WB? Please also show the individual data points and indicate the statistical test used. Please indicate in the legend what that bortezomib is a pan proteasomal inhibitor and bafilomycin A a lysosomal inhibitor for the general reader. Information about the concentration and timing of inhibitor application should also be included.

The two bands for the LMX1B^{H77L} protein can be best seen in Figure 3a, regrettably the resolution in panels b and c is not as high as in panel a. Thank you for pointing out the mislabeling of the bar graphs in panels b and c, this is now corrected. As requested, individual data points are shown and we mention that statistical significance was tested by an unpaired Student's *t*-test. We further explain that bortezomib is a pan-proteasomal inhibitor and that bafilomycin A_1 is a lysosomal inhibitor. We already mentioned that the inhibitors were added

together with cycloheximide, the concentration had been mentioned in the Materials section but is now also mentioned in the legend.

(Major point 4) Fig.4. Are the same samples shown on both membranes in panels a and b?

Yes, we are showing the same lysates and precipitates as now mentioned in the figure legend.

(Major point 5) Fig. 6. Please include an experimental scheme that reflects the timing of the interventions performed in the mice as they remain largely unclear when reading the result text and figure legend. How old were they at the beginning of the experiment, for how long was the treatment performed? How long was the wash-out period? Was all residual Lmx1b protein lost at the time of proteinuria measurement? How soon after doxycycline administration did the mice start developing proteinuria? It seems the whole intervention (dox+bort) lasted only 7 days in total?

The experimental time course is now shown. Seven to 10 week old mice were used for the experiments. There was no wash-out period, doxycycline was present in the drinking water throughout the experiment, bortezomib and DMSO were injected every other day for a total period of 6 days.

As we have shown by immunofluorescence staining for Lmx1b in the proteinuric mice (Fig. 7), there is still Lmx1b present in some podocytes. This is expected because it has been shown that the *NPHS2* promoter is not active in 100% of podocytes. Obviously it is sufficient for proteinuria to develop if the large majority of podocytes is affected.

Mice typically develop proteinuria earliest at 5 days after doxycycline administration, but reliably only after 7 days. Therefore the experiment only lasted for 7 days before the mice were killed because we wanted to avoid secondary effects from bortezomib treatment as much as possible.

As appropriate, these facts are now mentioned in the legend.

(Major point 6) Fig. 8. For the general reader, a minimal labeling of the EM pictures is advisable.

This has been done.

(Major point 7) Fig. 9. How old were the mice at the beginning of the experiment? Please show the PCA analyses of the datasets. Were podocyte-specific transcripts identified?

Mice were between 7-10 weeks old, this is now mentioned in the legend. Podocyte-specific transcripts were not identified.

(Major point 8) Please rephrase: "When bortezomib was administered to Lmx1b+/lox mice every other day, no obvious difference was detected to the DMSO-treated Lmx1b+/lox animals (Fig. 10a), corroborating the notion that bortezomib showed no nephrotoxic effect" as the presented data are not suitable for the assessment of nephrotoxicity.

This has been done.

(Major point 9) Fig. 10. Please show qPCR validation of the putative Lmx1b specific genes and validate an effect of their transcript regulation for the respective protein levels in podocytes (WB or IF).

Immunofluorescence stainings of selected proteins representing the four different clusters were performed with suitable antibodies, the expression of additional genes was analyzed by qPCR. Accordingly, the respective genes are now pointed out in the volcano blot in Fig. 9 and previous Fig. 10b has been replaced by the immunofluorescence stainings (Fig. 11) and the qPCR data (Fig. 12). By those two approaches, we have mostly been able to validate the expression patterns predicted from the cluster analysis.

(Major point 10) Suppl. Fig 4: Please show the individual data points.

This has been done.

(Major point 11) Suppl. Fig. 5: The graphs are labeled wrong (4h / 4h instead of 4h / 8h)? Please also show the individual data points and indicate the statistical test used. Please indicate in the legend what that MG132. Information about the concentration and timing of inhibitor application should also be included.

Thank you for pointing out the mislabeling of the bar graphs in panels b and c, this has been corrected (now suppl. Fig. 7). Individual data points are shown. The statistical test, the concentration of MG132 and the time of application are now mentioned in the legend.

(Minor point 1) Page 3 Introduction: Please clarify at the beginning of this text section, which discrepancies are being addressed. Between constitutive and podocyte-specific KO or between the patient mutations and the murine KO models (constitutive or inducible)?

This has been clarified.

(Minor point 2) Figure legend 1: Please write out „resp."

This has been done.

(Minor point 3) Suppl. Fig. 1: Please add a scale bar.

Regrettably, we did not include a ruler when the pictures of the paws were taken and therefore we are not able to provide a scale bar now. All we can say is that we did not notice any marked changes in the size of the paws.

(Minor point 4) Figure legend 4: Sentence unclear: „Only in the case of the two mutants, but not the wild-type, LMX1B proteins an additional smear can be detected in the presence of the HA-tagged ubiquitin chains.“

This has been done.
